# Metagenomic insights into taxonomic and functional patterns in shallow coastal and deep subseafloor sediments in the Western Pacific

**DOI:** 10.1099/mgen.0.001351

**Published:** 2025-03-18

**Authors:** Jiarui Sun, Miho Hirai, Yoshihiro Takaki, Paul N. Evans, Takuro Nunoura, Christian Rinke

**Affiliations:** 1 School of the Environment, The University of Queensland, St. Lucia, QLD, Australia; 2 Institute for Extra-cutting-edge Science and Technology Avant-garde Research (X-star), Japan Agency for Marine-Earth Science & Technology (JAMSTEC), Yokosuka, Kanagawa, Japan; 3 Australian Centre for Ecogenomics, School of Chemistry and Molecular Biosciences, The University of Queensland, St. Lucia, QLD, Australia; 4 Research Center for Bioscience and Nanoscience (CeBN), Japan Agency for Marine-Earth Science and Technology (JAMSTEC), Yokosuka, Japan; 5 Department of Microbiology, The University of Innsbruck, 6020 Innsbruck, Austria

**Keywords:** DNA extraction, deep biosphere, deep subseafloor, marine sediment, metagenomics, Pacific

## Abstract

Marine sediments are vast, underexplored habitats and represent one of the largest carbon deposits on our planet. Microbial communities drive nutrient cycling in these sediments, but the full extent of their taxonomic and metabolic diversity remains to be explored. Here, we analysed shallow coastal and deep subseafloor sediment cores from 0.01 to nearly 600 metres below the seafloor, in the Western Pacific Region. Applying metagenomics, we identified several taxonomic clusters across all samples, which mainly aligned with depth and sediment type. Inferring functional patterns provided insights into possible ecological roles of the main microbial taxa. These included *Chloroflexota*, the most abundant phylum across all samples, whereby the classes *Dehalococcoida* and *Anaerolineae* dominated deep-subsurface and most shallow coastal sediments, respectively. *Thermoproteota* and *Asgardarchaeota* were the most abundant phyla among Archaea, contributing to high relative abundances of Archaea reaching over 50% in some samples. We recovered high-quality metagenome-assembled genomes for all main prokaryotic lineages and proposed names for three phyla, i.e. *Tangaroaeota* phyl. nov. (former RBG-13-66-14), *Ryujiniota* phyl. nov. (former UBA6262) and *Spongiamicota* phyl. nov. (former UBA8248). Metabolic capabilities across all samples ranged from aerobic respiration and photosynthesis in the shallowest sediment layers to heterotrophic carbon utilization, sulphate reduction and methanogenesis in deeper anoxic sediments. We also identified taxa with the potential to be involved in nitrogen and sulphur cycling and heterotrophic carbon utilization. In summary, this study contributes to our understanding of the taxonomic and functional diversity in benthic prokaryotic communities across marine sediments in the Western Pacific Region.

**Impact Statement**

The exploration of marine deep-subsurface sediments is still in its infancy, mainly due to technological limitations such as the complexity and high costs of deep-sea drilling operations. This has led to a gap in our understanding of the diversity and metabolic abilities regarding the unique life forms inhabiting this ecosystem. In this study, we analysed microbial communities from deep subseafloor cores from the Western Pacific Region, recovered from sediments as deep as ~600 metres below the seafloor, and compared them to shallow coastal sediment samples. Using metagenomics, we explored the key environmental drivers of sediment microbial diversity, identified taxonomic and functional groupings, reconstructed metagenome-assembled genomes and assessed their main metabolic potential. Our results also demonstrate that deep-subsurface sediments harbour many novel and uncharacterized microbial lineages, a finding that can inform biodiversity conservation and the search for new biotechnological applications.

## Data Summary

All sequencing data have been deposited with the National Center for Biotechnology Information (NCBI) under multiple BioProjects: PRJDB12054 (off Shimokita Peninsula), PRJNA678545 (Sunshine Coast Lakes) and PRJNA678552 (Hikurangi Subduction Margin). Supplementary data, phylogenetic tree files and R scripts used in this study can be found via the journal’s FigShare portal with https://doi.org/10.6084/m9.figshare.28342124.v1 [1].

## Introduction

Marine sediments represent one of the largest contiguous ecosystems and have been estimated to hold up to 5.39×10^29^ cells, representing ~3.6% of the total living biomass on our planet [2]. Microbial communities in these sediments are highly diverse, with a taxonomic richness comparable to topsoil [3]. A range of abiotic factors contributes to this high diversity such as temperature, pH, pressure and the availability of nutrients and electron acceptors [3,9]. In addition, sedimentation rates, sediment consistency and porosity can influence microbial community compositions [3,9]. All these geochemical factors contribute to the observed spatial heterogeneity of marine microbial sediment communities; however, an overall pattern has emerged for subseafloor sediments, showing that microbial biomass and diversity are decreasing with sediment depth, due to diminishing energy availability [3,10,12].

Microbiomes play key roles in the biogeochemical cycling of nutrients such as carbon, nitrate, ammonium, phosphate and sulphur in marine sediments. One of the most important functions is the microbial oxidation, i.e. decomposition, of organic matter [13]. Every year, ~100 million metric tonnes (0.1 Pg) of organic matter is deposited in marine sediments [14], making these sediments one of the most expansive carbon reservoirs on our planet [15]. Microbes harness energy by mineralizing organic carbon through heterotrophic decomposition and further degradation through a series of redox reactions [16]. Under oxic conditions, aerobic respiration is used for carbon mineralization. However, oxygen availability decreases with sediment depth; e.g. in organic-rich sediments, oxygen concentration usually drops below detection limits within centimetres [17,18]; therefore, alternate terminal electron acceptors are required. In oxygen-deprived layers, overall nitrate, manganese (Mn IV) and iron (Fe III) reductions prevail closer to the sediment surface, whereas electron acceptors with lower energy yields such as sulphate and carbon dioxide, utilized for sulphate reduction and methanogenesis, respectively, dominate in deeper sediment layers [19]. Microbial communities were found to differ significantly between sulphate reduction and methanogenic zones, e.g. in South China Sea sediments [20]. However, sulphate-reducing bacteria were also reported from below the sulphate–methane transition zone (SMTZ), suggesting that the SMTZ is not a strict metabolic division in deep subseafloor sediments [21].

Several archaeal and bacterial lineages are found to dominate marine sediments, whereby a distinct taxonomic composition has been observed for oxic compared with anoxic sediment layers. For example, a recent PCR-based global survey of microbial community structures in marine sediments found that among Archaea, *Thermoproteota* (former ‘*Crenarchaeota*’) in particular the class *Bathyarchaeia* and the phyla *Asgardarchaeota*, *Hadesarchaeota* and *Nanoarchaeota* (a name that was proposed to be replaced with *Nanobdellota* [22]) were abundant in anoxic sediments, whereas the class *Nitrososphaeria* (former ‘*Thaumarchaeota*’) dominated in oxic sediment layers [3]. Within the domain Bacteria, anoxic sediments were dominated by *Atribacterota*, *Chloroflexota* and *Planctomycetota*, whereas *Alphaproteobacteria*, *Betaproteobacteria* [this lineage has been united with the class *Gammaproteobacteria* in the phylum *Pseudomonadota* in Genome Taxonomy Database (GTDB)] and *Firmicutes* (currently named *Bacillota*) were prevalent in oxic sediments [3]. Recent amplicon analyses of anoxic sediments report similar dominant bacterial lineages, for example, *Atribacterota*, *Dehalococcoidia* (phylum *Chloroflexota*) and *Aerophobota* in deep-sea samples from the South China Sea [20]; *Elusimicrobia* (phylum *Elusimicrobiota*), *Chloroflexota*, *Aerophobota* and *Lokiarchaeia* (phylum *Asgardarchaeota*) in the Costa Rica Margin subseafloor [23]; and *Atribacterota*, *Dehalococcoidia* and *Aminicenantes* in the South Atlantic [24].

Over the last decade, culture-independent methods, such as metagenomics and single-cell genomics, have allowed insights into the metabolic capabilities of the prevalent bacterial and archaeal lineages in marine sediments. For example, a metagenomic study of anoxic subseafloor clay in the abyssal North Atlantic concluded that members of the bacterial phylum *Atribacterota* are capable of performing homoacetogenesis, by oxidizing hydrogen to reduce carbon dioxide to acetate [25]. This lineage was also inferred to degrade a diverse range of organic matter compounds, driving heterotrophic decomposition in this ecosystem [25]. Similarly, a single-cell genomic study proposed a homoacetogenic lifestyle for the dominant *Chloroflexota* clade in deep-sea sediments of the Peru Margin [26]. Among Archaea, metagenome-assembled genomes (MAGs) of *Lokiarchaeia* recovered from sediments of an active vent field in the North Atlantic were inferred to be heterotrophic, consuming aromatic hydrocarbons while requiring sulphate-reducing bacteria as syntrophic partners [27,28]. Metagenomics also revealed that *Nitrososphaeria* were dominant in seafloor sediments near the surface in the South Atlantic and Central Pacific, presumably oxidizing ammonia with oxygen as the electron acceptor [24,29].

Despite these forays into community profiling and metabolic reconstructions of microbes in marine sediments, a great number of these habitats, especially deep-sea subsurface layers, have remained unexplored. Here, we aim to understand the distribution patterns of marine sediment communities by comparing shallow coastal and deep subseafloor samples and associated chemico-physical metadata. Applying metagenomics allowed us to recover taxonomic clusters, to identify functional groupings across sites and to assign these functions to MAGs of archaeal and bacterial lineages, in order to predict their ecosystem relevant roles. The recovery of genomes from three uncharacterized lineages leads to the proposal of the new phyla *Tangaroaeota* phyl. nov. (former name RBG-13-66-14), *Ryujiniota* phyl. nov. (UBA6262) and *Spongiamicota* phyl. nov. (UBA8248). This study represents the first community-wide metagenomic exploration of four geographically distinct sites, located in the West Pacific Region.

## Methods

### Site description, sampling and geochemistry

#### Shimokita Peninsula sediment

Northwest Pacific forearc basin sediments were taken from site C9001 [also cited as International Ocean Discovery Program (IODP) C0020] located off the Shimokita Peninsula (SP), Japan (41° 10.638' N, 142° 12.081' E, water depth of 1180 m), ~80 km east off the coast. Sediment cores totalling 365 m were recovered by using a hydraulic-piston coring system from hole C9001C by the drilling vessel (DV) Chikyu during JAMSTEC CK06-06 cruise in 2006 [30,31]. Whole round cores (WRCs) with 10 cm in length were taken for molecular biological analyses and stored at −80 °C. Previous studies have reported profiles of lithology, age model, porewater inorganic chemistry, organic chemistry, cell abundance and microbial community compositions in sediments at site C9001 [30,35].

#### Hikurangi Subduction Margin sediment

Deep-sea sediments from Hikurangi Subduction Margin (HSM) were sampled from four sites (U1518, U1519, U1520 and U1526) during the IODP Expedition 375 using DV *JOIDES Resolution* in 2018 [36]. These sampling sites are located at an active fault near the deformation front (U1518), the upper plate above the high-slip slow-slip event source region (U1519), the incoming sedimentary succession in the Hikurangi Trough (U1519) and atop the Tūranganui Knoll Seamount (U1526), at coordinates between 38°43'39.4"S, 178°36'53.7"E and 39°1.3204′S, 179°14.7594′E. Details on drilling at these sites was provided by Saffer *et al*. [36]. Onboard sampling of the WRCs was carried out using 5-ml syringes, with the syringe top and the luer lock cut off so that the syringes could be used to recover a small subcore from the WRCs. The subcore samples were stored in a −86 °C freezer and shipped on dry ice until they reached the laboratory, where they were stored at −80 °C until DNA extraction [37]. In this study, we investigated 11 sediment samples of U1519 at depths from 1.5 to 280.17 metres below the seafloor (mbsf), 10 sediment samples of U1520 at depths from 3.03 to 1006.84 mbsf and 2 samples of U1526 sediments at depths of 0.01 and 20.32 mbsf, to explore the microbial communities at this area (Table S1a, available in the online Supplementary Material).

#### Sunshine Coast Lakes sediment

Lake sediment samples from Lake Cootharaba (LC) (26°16'49" S, 152°59'24" E) and Lake Weyba (LW) (26°26'24" S, 153°3'36" E) were sampled using sterilized 1-m Polyvinyl chloride (PVC) pipes. The sediments at these two lakes consisted of yellow, loose sand at the surface that gradually turned into black sediment at ~10 cm. At around 20–40 cm depth (varied in different cores), the sediments started transferring into dense clay in grey or dark grey colours. The measured physicochemical data for water samples from LW and LC (Table S1b) suggested that the former is a hypersaline lake, whereas the latter is a saline lake, according to water salinity classification (Department of Water, Government of Western Australia). LC sediments at depths from 5 to 25 cm and LW sediments at depths from 5 to 60 cm were subsampled from PVC cores in 5-cm intervals in December 2018 and November 2019, which has been described in our previous study together with chemical analysis [37]. Besides, two sediment samples at depths 20–30 and 30–40 cm from one additional core from LW were subsampled using 5-ml syringes (with the hub and seal removed, then sterilized using bleach, alcohol and UV for 1 h) in December 2020. The collected samples were flash frozen in liquid nitrogen and a subset was delivered to ALS Environmental Testing, Brisbane, Australia, for chemical analysis. The remaining samples were transported directly to the laboratory and stored at −80 °C for further analysis. In total, 11 and 7 sediment samples from LW and LC were analysed, respectively (Table S1a).

### DNA extraction and metagenomic sequencing

A total of 12 representative WRCs from the Northwest Pacific site (SP), at depths of 0.9, 9.3, 18.5, 30.8, 48.3, 59.5, 68.8, 87.7, 116.4, 154.3, 254.7 and 363.3 mbsf, were selected and used in this study. Detailed sample descriptions were provided previously [38]. Notable geochemical and lithological features of these samples are summarized here, listed by depth: at 0.9 mbsf, the uppermost section of the core column was identified as sulphate reduction zone; at 9.3 mbsf, the sediment represented the region just beneath the SMTZ; at 68.8 mbsf, the sediment had anomalously high concentrations of coenzyme F430, a biomarker of methanogenesis [32]; and at 116.4 mbsf, the sediment consisted of ash/pumice, whereas all other SP samples used in this study were pelagic clay sediments [21,32]. Total environmental DNA was extracted from ~5 g of sediment. Details of DNA extraction and shotgun metagenomic library construction are described in Hiraoka *et al*. [39] and Hirai *et al*. [40] and summarized in Medvedeva *et al*. [38].

For the HSM and Sunshine Coast Lake sediments, 28 and 18 samples, respectively, were used in this study. To minimize possible contamination, the outer centimetre of each sediment subsample was trimmed off with sterile blades to use only the inner sediment core for DNA extraction. DNA was extracted from the pre-processed sediment samples using Mo BIO’s PowerSoil DNA Isolation kit (QIAGEN, USA) following the manufacturer’s protocol with minor modifications in bead beating and DNA eluting steps. Around 300 mg sediments were mixed with 0.25 ml G2 DNA/RNA Enhancer 0.1 mm beads (Ampliqon, Denmark) and 650 µl bead solution (QIAGEN) (Table S1a), using Mo Bio’s PowerLyzer 24 Homogenizer (QIAGEN) under 4000 g for 45 s. The extracted DNA was eluted from the spin filter using 30 µl UltraPure™ DNase/RNase-Free Distilled Water (Thermo Fisher Scientific, USA), and this step was repeated by adding the same water (that now contained the eluted DNA) onto the spin filter one more time to maximize the DNA elution. This approach yielded at least 20 µl of DNA eluate. Concentrations of recovered DNA were quantified using Qubit 2.0 Fluorometer (Thermo Fisher Scientific; Table S1). Illumina Nextera XT libraries were prepared using a low-input DNA library protocol [41] and pair-end sequenced on the NextSeq 500/550 High Output v2 system with 150 bp read length.

### Assembly, binning and taxonomic assignment

Shotgun-sequenced raw reads were processed using SeqPrep (https://github.com/jstjohn/SeqPrep) under default settings to merge overlapping paired-end reads and trim adaptors. Metagenomic assembly and binning were performed using the Aviary pipeline [42]: pre-processed reads (post-SeqPrep) were first assembled with metaSPAdes [43], then Minimap2 [44] and Samtools [45] were used to map sequences back to the assemblies and CoverM [46] was used to generate coverage reports. Multiple tools were used in the binning step of Aviary pipeline, including CONCOCT [47], VAMB [48], MetaBAT [49], MetaBAT2 [50], MaxBin2 [51], Rosella [52] and DASTool [53].

CheckM2 [54] was applied to calculate the statistics of bins recovered from the Aviary pipeline. MAGs with completeness of >50% and contamination of <10% were kept as the final genome sets. Taxonomy was assigned to MAGs using GTDB-Tk v2 [55] against GTDB (RS214) based on relative evolutionary divergence values. Phylogenies of recovered MAGs were analysed using FastTree [56] (for bacterial MAGs) or using FastTree and IQ-TREE [57] (for archaeal MAGs) to confirm the assigned taxonomy. To further confirm the taxonomy of the three novel bacterial phyla (o__RBG-13-66-14, p__UBA6262 and __UBA8248), sequences of 1654 order-level representative genomes together with 686 bacterial MAGs were subsampled from the multiple sequence alignment used in the above bacterial genome tree. Maximum-likelihood phylogenies were inferred using FastTree [56] and IQ-TREE [57]. After re-root and decoration with GenomeTreeTk (https://github.com/donovan-h-parks/GenomeTreeTk) and PhyloRank (https://github.com/donovan-h-parks/PhyloRank), the consensus tree was analysed in arb [58].

### Identification and annotation of functional core genes

ORFs were predicted from both the assembly contigs and MAGs using Prodigal v2.6.3 [59] with the extensions ‘--metagenome’ and annotated with KofamScan [60] using KEGG release 95.0 (https://www.kegg.jp/kegg/kegg2.html). Only protein sequences with scores above the Kofam predefined thresholds were used to explore functionalities.

To discriminate the reductive and oxidative types of the encoded *α*-subunits of the dissimilatory sulphite reductase (DsrA), we used 19 sequences from the DsrA aa sequence database in Müller *et al*. [61], which includes 3 reductive archaeal type DsrA (PrbAero3, CvgMaqu3 and entry24y), 7 reductive bacterial type DsrA (FP929047, ArgVene3, PtmSpec4, DslSp133, DscApsh6, TdsIsla2 and TdlAtlan), 5 oxidative bacterial DsrA (ClrChlo9, MagGryp3, SfcDeni2, MarPurpu and UnSYyyyy) and 4 unclassified DsrA sequences (MooThe10, entry669, entry674 and MooTher5) that were used as outgroup. Protein sequences derived from assemblies and MAGs assigned to K11180 (dissimilatory sulphite reductase alpha subunit) were collected and aligned with MAFFT v7.455 [62] together with the selected reference DsrA genes. The alignment was then trimmed by TrimAl v1.495 [63] with ‘-gt 0.9 -cons 60’ selection. To identify *mcrA* types, genes from MAGs and metagenome assemblies were first screened with the McrA package provided by GraftM [64]. Protein sequences of the detected *mcrA* genes, together with 46 reference *mcrA* genes collected from Timmers *et al*. [65], were aligned and trimmed with MAFFT v7.455 [62] and TrimAl v1.495 [63], respectively. Maximum-likelihood trees of both DsrA and McrA were calculated by FastTree [56] with ‘-wag -gamma’, which was then used to calculate the final tree in IQ-TREE [57] under ‘LG+C60+F+G+PMSF’ model with 1000 ultrafast bootstrap replicates. All phylogenetic trees inferred in this study were viewed and annotated by iTOL [66].

### Taxonomic profiling and statistical analysis

To obtain microbial community profiles, we applied a single-copy marker gene-based approach using singleM v0.15.0 [67], a tool for profiling shotgun metagenomes, against the GTDB (RS214) [68]. SingleM detects 35 bacterial and 37 archaeal single-copy marker genes and calculates relative abundances of marker gene-based operational taxonomic units (mOTUs) based on read coverages. While our lab procedure was designed to minimize possible contamination (see the section ‘DNA extraction and metagenomic sequencing’), we also investigated instrument, reagent or operator-introduced contamination in a manual quality control step. To detect these contaminants, we took advantage of the facts that they are generally found in negative controls, usually appear at elevated frequencies in low-input DNA samples [69], and that many contaminants associated with laboratory reagents, human operators and the laboratory environment have been identified [70]. Based on these three parameters, we screened our HSM, LC and LW community profile data and removed a total of seven taxa identified on the family and/or genus level: f__*Propionibacteriaceae*, f__*Nocardioidaceae*, g__*Streptococcus*, f__*Beijerinckiaceae*, f__*Treponemataceae*, g__*Corynebacterium* and g__*Staphylococcus*. For SP samples, we screened the contaminant taxa that were identified in the previous study [21], and none of them were detected.

After removing contaminant mOTUs and mOTUs with low abundances, i.e. mOTUs with maximum relative abundances <1% across all samples, statistical analyses of community profile were performed in RStudio software v1.1.456 [71] using basic R packages and ‘vegan’ and visualized with RStudio software or Prism9 (https://www.graphpad.com/). The Shapiro–Wilk normality test was conducted to confirm the normal distribution of the 53 samples. The estimated species richness of the 18 lake and 35 marine sediment metagenomes was evaluated with package ‘breakaway’. Rarefaction curves and alpha diversity indices (Shannon, Abundance-based Coverage Estimator also known as ACE, and Chao1) were calculated by functions ‘rarefy’, ‘rarecurve’, ‘diversity’ and ‘estimateR’ in vegan package. ANOVA with Tukey’s HSD post hoc analysis was calculated to determine the significant differences in species compositions among sampling sites.

Beta diversity analyses with dissimilarity based on Euclidean distances were performed on the Hellinger-transformed mOTU table using ‘decostand’ and ‘vegdist’ functions. To study the effects of environmental factors on the SP, HSM, LW and LC community profiles, redundancy analyses were conducted based on the Euclidean distances with the function ‘rda’, ‘dbrda’ or ‘wcmdscale’. To determine the significant variations in prokaryotic diversities in terms of environmental factors, the permutational multivariate ANOVA (PERMANOVA) [72] was performed using ‘adonis2’. Only significant parameters through a forward model selection were fitted to the ordination plots with ‘envfit’.

Statistical analysis of microbial functional profile was based on the gene counts of identified KEGG Orthologs in metagenomes, which were normalized with DESeq2 [73] and visualized with ‘pheatmap’ in R. A principal component analysis was then performed and visualized using functions ‘vst’ and ‘plotPCA’ of ‘DESeq2’.

## Results

### Description of sampling locations

**Fig. 1.**
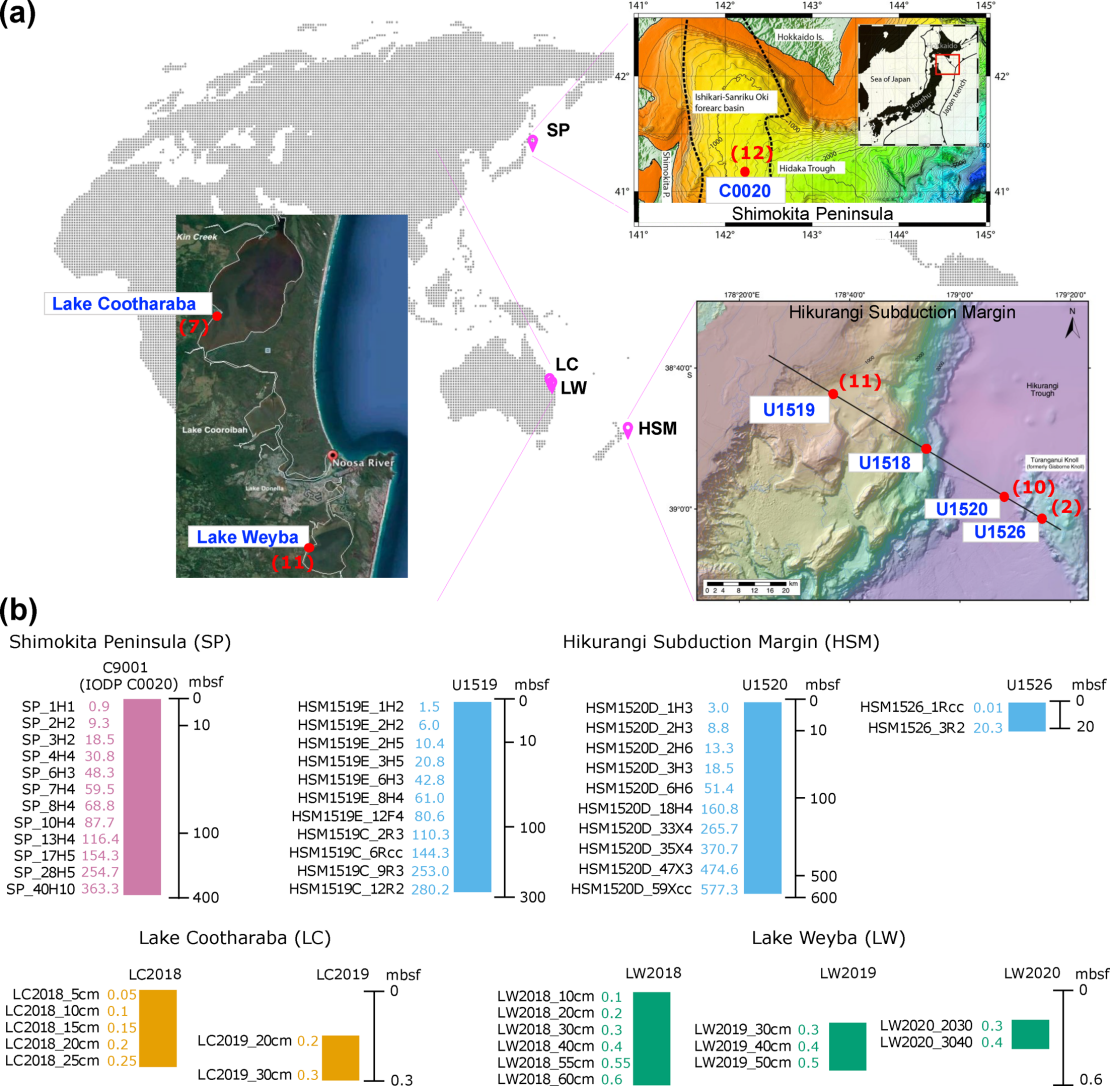
Sampling site descriptions.** (a)** Global locations of sampling sites. The bathymetric map of SP site C9001 (IODP site C0020) was modified from Figure F1 of ‘IODP Expedition 337 Preliminary Report’ [110]. The bathymetric map of HSM was modified from Figure F3 of ‘IODP Expedition 375 Preliminary Report’ [36]. The satellite image of LC and LW was modified from Skilleter *et al*. Red dots indicate sampling locations, and the number of samples sequenced is provided in red font next to each site. (**b)** Sediment samples analysed in this study. Holes from all sampling sites are shown and colour coded by site. Sampling depths are provided on the left side of each hole. Note that the SP hole C0020 and the LC and LW holes resulted in a single core each, whereas samples from HSM sites U1519, U1520 and U1526 were obtained from three, two and one cores, respectively. See Fig. S1 for detailed HSM core descriptions.

Our study comprised a total of 53 marine and coastal sediment core samples ranging from 0.01 to 577.3 mbsf (Fig. 1, Table S1). We analysed samples from a deep subseafloor site (C0020) located off the SP in Japan, from three deep subseafloor sites (U1519, U1520 and U1526) at the HSM off the coast of New Zealand and from two shallow coastal sites, LC and LW in Queensland, Australia (Fig. 1). For each site, we obtained sediment type characterizations, revealing loose sand and dense clay layers at the shallow coastal sites and mud, silt, clay and sand- and mudstone layers at the deep subseafloor sites (Text S1).

### Microbial diversity and responses to environmental factors

**Fig. 2.**
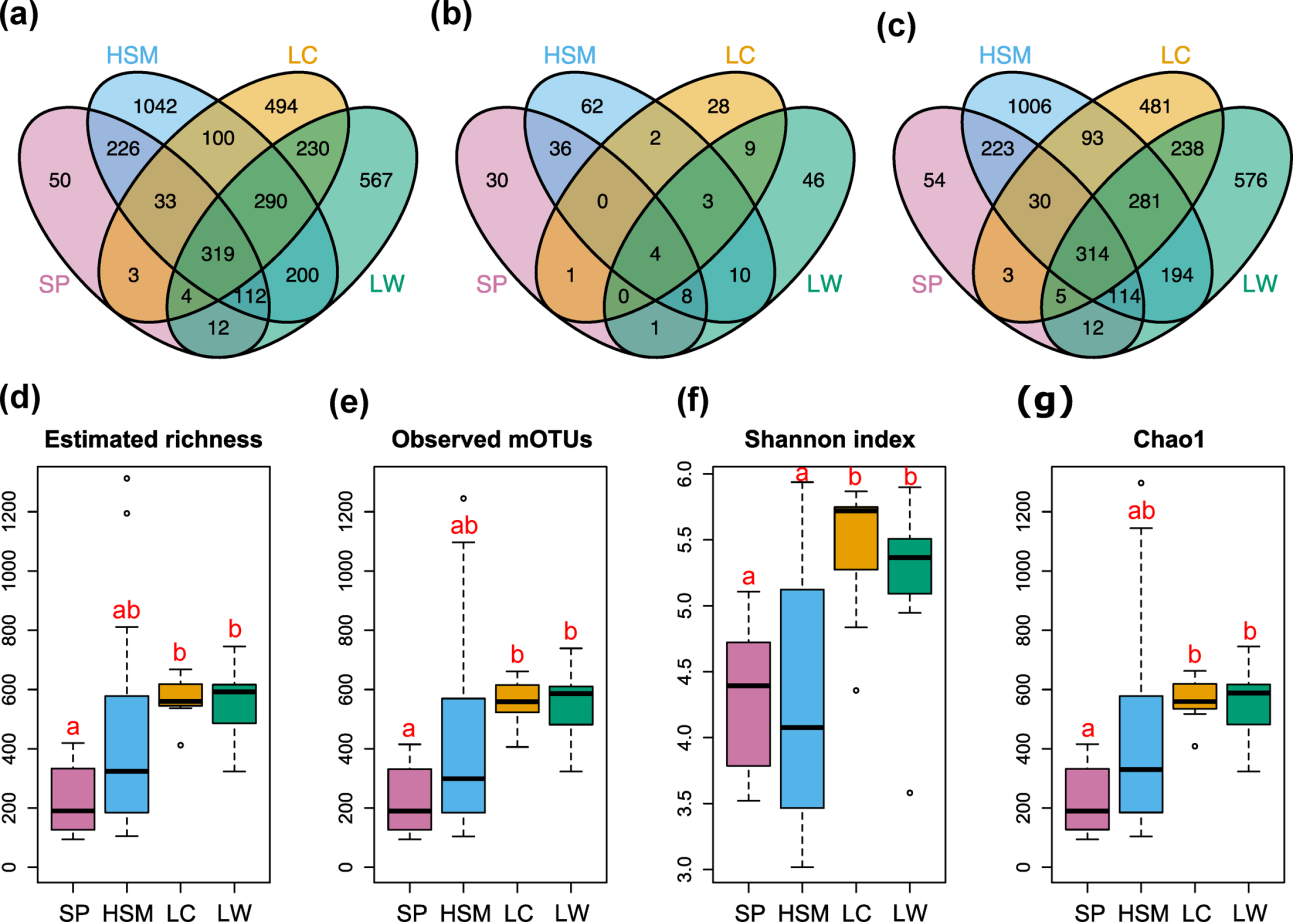
Species richness of mOTUs. (a) Venn diagram of all mOTUs. (**b)** Venn diagram of abundant mOTUs (i.e. mOTUs≥1% in at least one sample, [111]). (**c)** Venn diagram of rarefied mOTUs (rarefied to 26 967). (**d)** Species richness of estimated microbial communities among sampling locations. (**e)** Observed taxa of rarefied microbial communities among sampling locations. (**f, g)** Alpha diversities of rarefied observed microbial communities among sampling locations. The letters above the bars indicate significant differences (*P*<0.05) between sampling locations according to ANOVA with Tukey post hoc tests.

To explore microbial community structures, we recovered mOTUs from our shotgun data using a recently developed, single-copy marker gene approach. Following quality control, a total of 3827 prokaryotic mOTUs were detected across all samples (Table S2), of which 50, 1042, 494 and 567 abundant mOTUs were unique to SP, HSM, LC and LW sediments, respectively (Fig. 2a). Among the 240 abundant mOTUs, defined as having a relative abundance above 1% in at least 1 sample, a subset of 30, 62, 28 and 46 mOTUs were exclusively found in SP, HSM, LW and LC sediment samples, respectively (Fig. 2b). To account for different sequencing depths, we rarefied the data, and the results confirmed that SP samples had the lowest number of unique mOTUs (Fig. 2c, Table S3). Rarefaction curves suggested that we recovered the great majority of mOTUs present in the SP and HSM sites, whereas several LC and LW samples still harbour unexplored diversity (Fig. S2).

**Fig. 3.**
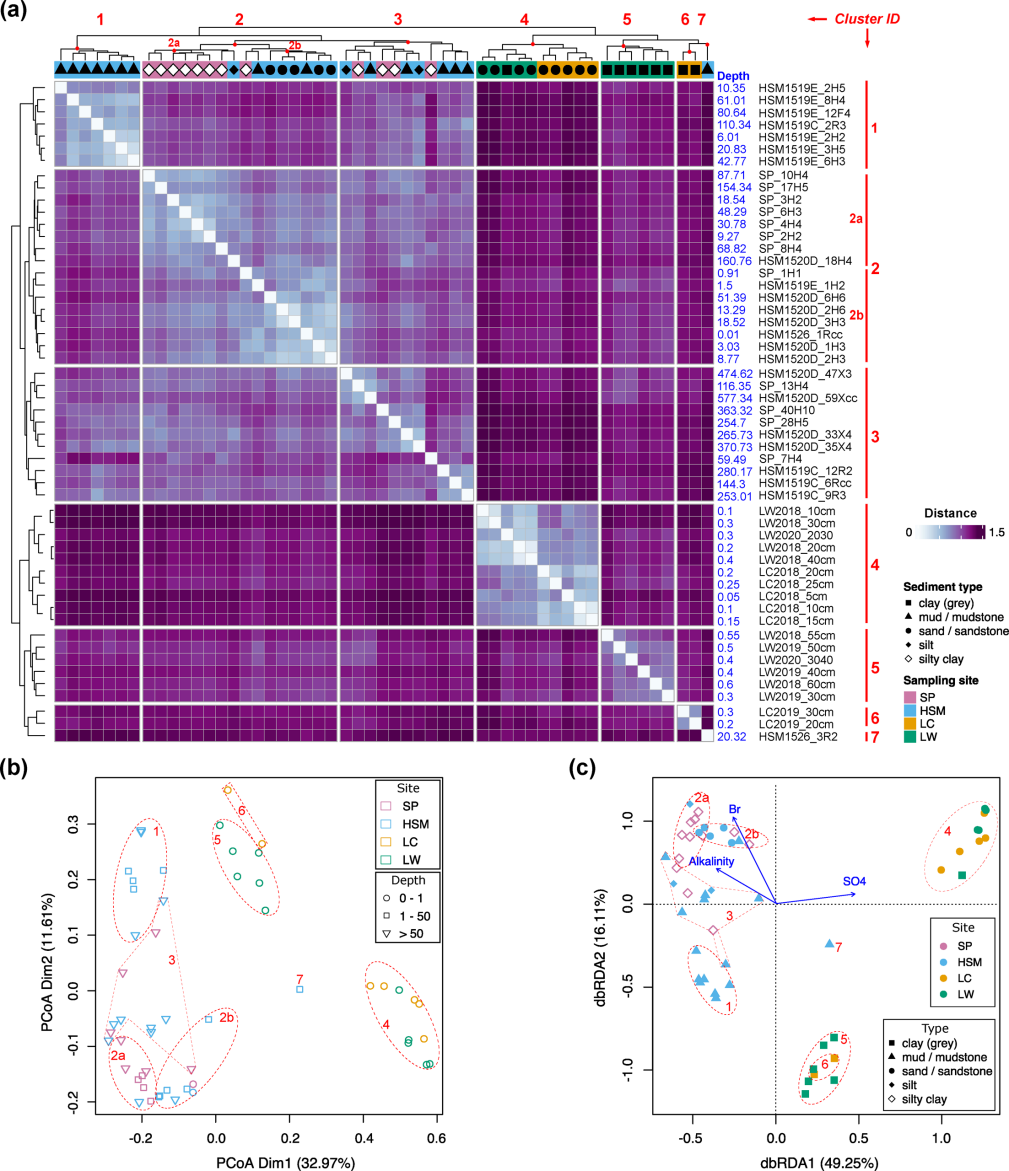
Beta diversity of prokaryotic mOTUs across samples. (a) Heatmap of Euclidean distance-based beta diversity. The heatmap was split into six major clusters with cluster numbers labelled on the top and left. Note the two subclusters of cluster 2 (2a, 2b). Sampling site and sediment type of each sample were annotated with different colours and shapes, respectively. The depth below the seafloor (depth) is provided for each sample. (**b)** Variance-based principal coordinate analysis (PCoA). (**c)** Distance-based principal component analysis, with environmental factors selected from stepwise PERMANOVA fitted to the first dimension with 5000 permutations. The sampling site and type of centroids were omitted. All three figures were based on the 240 mOTUs that had a maximum abundance of >1%.

Microbial community structures within and across the four sampling sites were compared via alpha and beta diversity. To assess alpha diversities, we first estimated mOTU richness, which was significantly higher in shallow coastal areas compared with deep subseafloor sediments (Fig. 2d). Diversity indexes and rarefaction curves confirmed this trend, with significantly higher species diversities in coastal sediments compared with deep subseafloor sediments (Figs 2e–g and S2). To estimate beta diversities, distance matrices of abundant mOTUs were constructed using Euclidean distance (Fig. 3a). Overall, microbial community compositions were strongly aligned with depths and sediment types. In deep-sea sediments, we found relatively high compositional similarity across the HSM and SP samples, which formed three clusters, labelled as clusters ‘1’, ‘2’ and ‘3’ in Fig. 3(a), which included samples taken from 0.01 to 57.34 mbsf. Cluster ‘1’, as well as subgroup ‘2a’ in cluster ‘2’, grouped mostly according to sediment type. The coastal samples, LW and LC, clustered distinctly from deep-sea samples but did not primarily group by site. Rather, samples from shallow sand layers (cluster ‘4’) were separated from the deeper clay layers (clusters ‘5’ and ‘6’; Fig. 3a). Ordination analyses confirmed the similarities of microbial communities between SP and HSM sediments and a clear separation among lake sediments according to sediment type (Fig. 3b, c).

Overall, microbial diversities showed strong correlations with sampling locations, depth and sediment type (all *P*<0.01; Table S4a). Microbial community compositions were also associated with alkalinity (*R*^2^=0.63, *P*=0.003), sulphate (*R*^2^=0.58, *P*=0.005) and bromine (*R*^2^=0.81, *P*<0.001), based on a constraint redundancy analysis (Fig. 3c, Table S4b). Multiple regression modelling using forward selection revealed factors shaping community structures at specific sampling locations, although with low *R*^2^ values. Iodine concentration (*R*^2^=0.23, *P*=0.001) and alkalinity (*R*^2^=0.20, *P*=0.004) correlated with SP samples (Fig. S3a, Table S4c), whereas concentrations of magnesium (*R*^2^=0.25, *P*=0.001) and sulphate (*R*^2^=0.09, *P*=0.022) were found to correlate with HSM samples (Fig. S3b, Table S4d). Additionally, LC and LW sediments were best predicted by pH (*R*^2^=0.38, *P*=0.035; Fig. S3c, Table S4e).

### Microbial community composition and taxonomy

**Fig. 4.**
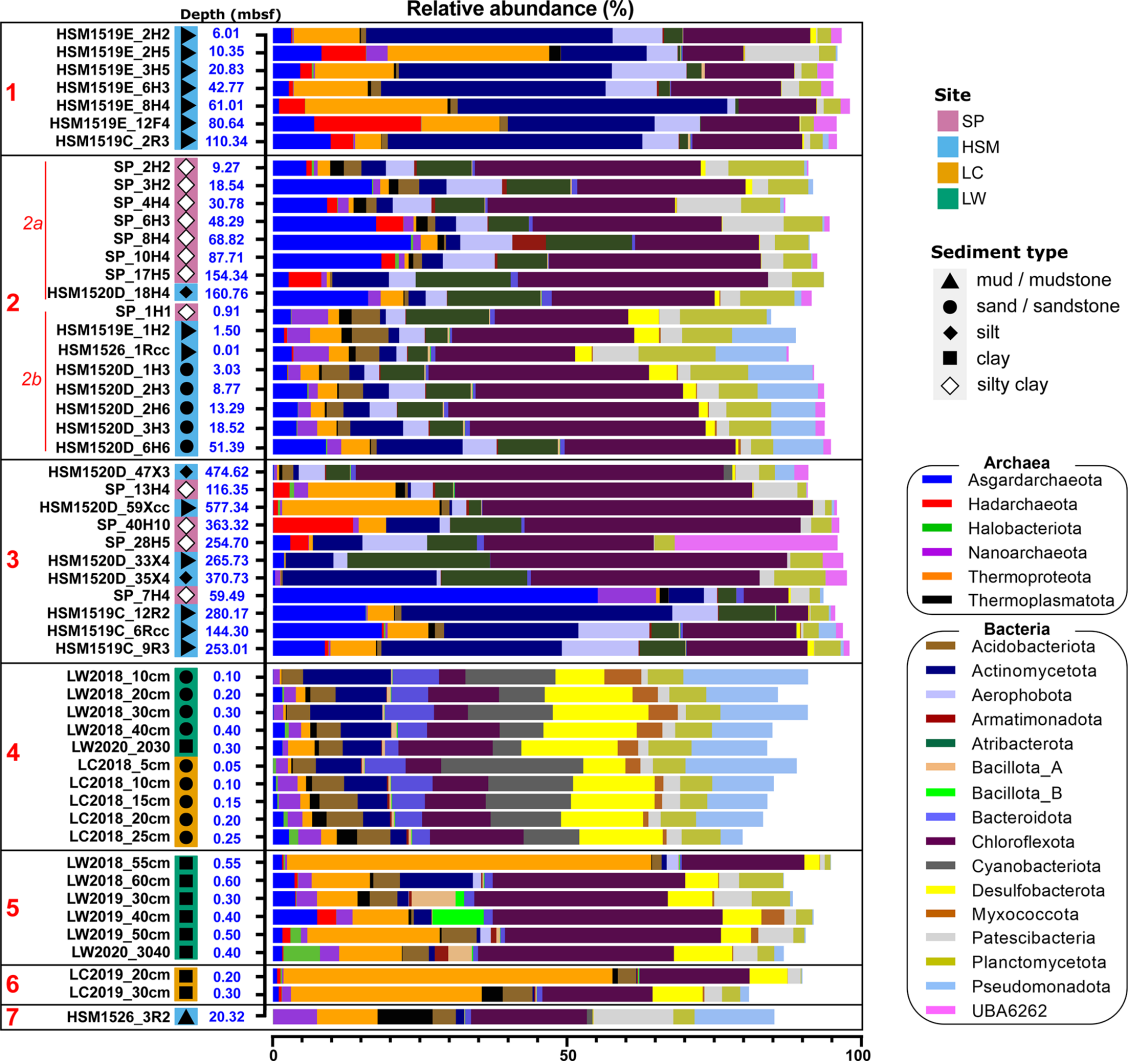
Phylum-level community profiles of SP, HSM, LC and LW samples. Marker protein-based community profiles. Twenty-two abundant phyla with maximum relative abundances greater than 5% are shown in this figure. Samples are ordered by taxonomic cluster, obtained through distance matrices of abundant mOTUs that were constructed using Euclidean distance (Fig. 3a). For detailed community profiles, see Tables S3 and S5. The figure was created with Prism9 (https://www.graphpad.com/).

Microbial community profiles in the deep subseafloor and coastal sediments were dominated by Bacteria, with an average relative abundance of 79.6±15.2% (Figs 4 and S4a, Table S5). However, in three samples (one SP, LC and LW sample; Fig. S5), Archaea made up the majority of the community, reaching up to 67.8%. Based on GTDB (RS214) taxonomy, the most abundant phylum, averaged across all samples, was *Chloroflexota* (26.0±14.0%), followed by *Actinomycetota* (10.9±13.1%), *Planctomycetota* (5.2±3.1%), *Atribacterota* (4.8±5.7%), *Pseudomonadota* (4.3±5.8%) and *Desulfobacterota* (4.1±5.3%) (Figs 4 and S4a, Table S5). In deep-subsurface samples, the vast majority (90.6±10.1%) of *Chloroflexota* were assigned to the class *Dehalococcoida*, in which members have been reported to perform anaerobic hydrogen oxidation [74], whereas in the shallow coastal sediments, mostly *Anaerolineae,* but in some samples also *Dehalococcoida*, dominated (Fig. S6). In the archaeal domain, *Thermoproteota* (8.5±12.7%) and *Asgardarchaeota* (6.0±9.0%) represented the phyla with the highest average relative abundances (Figs 4 and S4a, Table S5).

Assessing the community profiles based on the clusters derived from beta diversity comparisons (Fig. 3) revealed underlying taxonomic patterns (Fig. 4). For example, cluster 1, consisting exclusively of HSM site U1519, was dominated by *Actinomycetota*, ranging from 14.6 to 46.0% in relative abundance (Figs 4 and S4a). Within this phylum, the orders JAHJRV01 and *Humimicrobiales* in the class *Humimicrobiia* were most abundant (Fig. S7; Tables S3, S5 and S6). *Aerophobota*, mainly the family AE-B3A (order *Aerophobales*), were prevalent and reached relative abundances of up to 13.0%. Among Archaea, *Thermoproteota*, mainly the class *Bathyarchaeia*, dominated, and this phylum reached maximum relative abundances of 27.5%. *Hadarchaeota* were not as prevalent but reached high abundances in some samples, e.g. up to 18.1% in sample U1519E-12F4 (Fig. 4, Table S5).

Cluster 2, made up of HSM and SP samples, was dominated by *Chloroflexota* and had a strong presence of *Atribacterota* and *Asgardarchaeota* (Fig. 4). In subcluster 2a, *Asgardarchaeota*, mainly the classes *Lokiarchaeia* and *Heimdallarchaeia* (Table S5), were abundant at depths from 0.9 to 87.7 mbsf and reached up to 23.4% in sample (SP_8H4). At the shallowest sites in subcluster 2b, at 0.1 and 0.9 mbsf, samples HSM U1526-1Rcc and SP-1H1, *Nanoarchaeota* was the most abundant archaeal lineage with 6% and 6.2%, respectively. Within this phylum, most reads were assigned to an unclassified genus in the family GW2011-AR1 (order *Pacearchaeales*; Fig. S7).

Cluster 3 was composed of a more heterogeneous community, mostly dominated by *Chloroflexota*, mainly the class *Dehalococcoida*. In the deepest samples, at 474.6 and 577.4 mbsf, *Dehalococcoida* reached the highest proportions, accounting for up to 56.2% and 54.8% of the prokaryotic community (Table S5). *Asgardarchaeota* abundances were low, and the dominant archaeal taxon was the class *Bathyarchaeia* (phylum *Thermoproteota*), in particular the order B26-1 at the deepest site (HSM 15020D-59Xcc) at 577.3 mbsf (Figs 4 and S7; Tables S3, S5 and S6).

However, an exception was the subcluster comprised of the deepest samples from site U1519 with high relative abundances of *Actinomycetota*, but comparably lower *Thermoproteota* abundances compared with samples from the same site that formed cluster 1. Two SP samples, 28H5 and 7H4, were outliers in cluster 3 with high relative abundances of *Asgardarchaeota* and the previously uncharacterized phylum UBA6262, respectively. The high relative abundance of UBA6262, for which we propose the name *Ryujiniota* phyl. nov. (see the section ‘Description of novel phyla and type material’), of up to 27.7% at 254.7 mbsf at SP (sample 28H5) and its presence at other SP and HSM sites (Fig. 4, Table S5) combined with the absence of this lineage from our shallow coastal samples suggest that this phylum is adapted to the life in the deep subsurface.

Among the shallow sites, the sand sediment cluster 4 showed a rather diverse set of dominant phyla, including *Chloroflexota* (18.7±11.3%), *Desulfobacterota* (10.4±4.5%) and *Pseudomonadota* (7.2±7.1%). In the latter phylum, the order *Burkholderiales* (class *Gammaproteobacteria*) was highly abundant (Fig. S7, Tables S3 and S6). The shallower sandy layers also contained *Cyanobacteriota*, with a maximum relative abundance of 24.2% (Figs 4 and S4a, Table S5), comprised of several cyanobacterial families in the order *Cyanobacteriales*, including *Microcystaceae*, *Microcystaceae*_A and *Xenococcaceae* (Fig. S7, Table S3), and the family *Cyanobiaceae* in the order PCC-6307. Overall bacterial relative abundances decreased slightly with depth, whereas archaeal relative abundances increased to a maximum of 15.9% (Figs 4 and S5, Table S5).

The deeper clay layers of the shallow coastal sediments making up cluster 5 were mostly dominated by *Chloroflexota*, mainly by members of *Anaerolineae,* but *Dehalococcoidia* prevailed in some samples (Figs 4, S6 and S7). In the latter class, the most abundant genus was AB-539-J10 (family GIF9) with up to 8.9%. The same genus was also present in the deep subseafloor samples HSM 1520 and 1519, with a maximum abundance of 14.7% in HSM1520 at 51.4 mbsf (Fig. S7, Table S3). These results suggest a wide geographic range of the so far not described genus AB-539-J10. Another abundant bacterial phylum was *Desulfobacterota*, including the unexplored genus 0-14-0-80-60-11 (order *Desulfobaccales*; Fig. S7). Interestingly, two samples (LW2019_30 cm and LW2020_30 cm) were enriched in *Bacillota*_A (7.5%), mainly the family *Lachnospiraceae* (class *Clostridia*), and *Bacillota*_B (8.7%), in particular the genus *Desulfocucumis* (class *Desulfotomaculia*). Sulphate-reducing strains of *Desulfocucumis* have been reported from marine sediments [75], and the source of *Lachnospiraceae*, which are anaerobes found in the animal digestive tract of mammals and humans [76], could be the digestive tract and faeces of fish, as suggested previously [77]. Archaeal abundances were higher in this cluster compared with the shallow sand layers (cluster 4) and reached up to 29.4%, except for sample LW2018_55 cm, which was dominated by Archaea (64.9%). The great majority of these archaeal reads were assigned to the class *Bathyarchaeia* (phylum *Thermoproteota*), including the genus SOJZ01 (family BA1, order B26-1) (Fig. S7).

Cluster 6, formed by two LC clay samples, was dominated by the classes *Bathyarchaeia* (*Thermoproteota*) and *Dehalococcoidia* (*Chloroflexota*) (Table S5).

Cluster 7 consisted only of a single sample, HSM U1526-3R2, taken at 20.3 mbsf. Here, *Thermoproteota* dominated among Archaea, with 94.2% of all *Thermoproteota* reads assigned to the *Nitrososphaerales* families *Nitrosopumilaceae*, in particular to the genus DRGT01, and the uncharacterized family UBA57 (Tables S3 and S5). Among the bacterial lineages, the 20.3 mbsf sample was also quite unique and showed the highest relative abundances of *Patescibacteria* (13.6%), which were mainly assigned to the class *Paceibacteria* (Table S5).

Several phyla with placeholder names in the GTDB RS214 (https://gtdb.ecogenomic.org/), representing unnamed, uncultured lineages, occurred in a subset of samples. For example, phylum UBA8248, for which we proposed the name *Spongiamicota* phyl. nov., was only detected in LW2018_55 cm at 0.1%, whereas phylum QNDG01 was present in most LW samples and one LC sample with a maximum abundance of 0.63% (Table S5). Phylum RBG-13-66-14, for which we proposed the name *Tangaroaeota* phyl. nov., was widely detected at HSM site 1520 with a maximum abundance of 0.2%. Phyla SM23-31 and UBP18 showed a broader distribution across HSM, SP and LW samples with maximum abundances of 0.3% and 1.0%, respectively (Table S5). Phyla SZUA-182 and WOR-3 were recorded at 1.2% and 3.5%, but only in LC and SP samples, respectively. These results support previous findings that marine sediments hold great potential for the discovery of novel, uncharacterized lineages.

### Metagenome-assembled genomes

Through shotgun read assembly, binning and quality screening, we recovered a total of 905 MAGs (Table S7), with medium-to-high quality (completeness >50%, contamination <10%) [78]. Overall, the recovered MAGs had an average estimated completeness of 85.5±11.0% with an estimated contamination of 2.4±2.1%. The GC content ranged from 27.0 to 69.0%, and the average genome size was estimated to be 2.2±1.4 Mbp (Table S7).

**Fig. 5.**
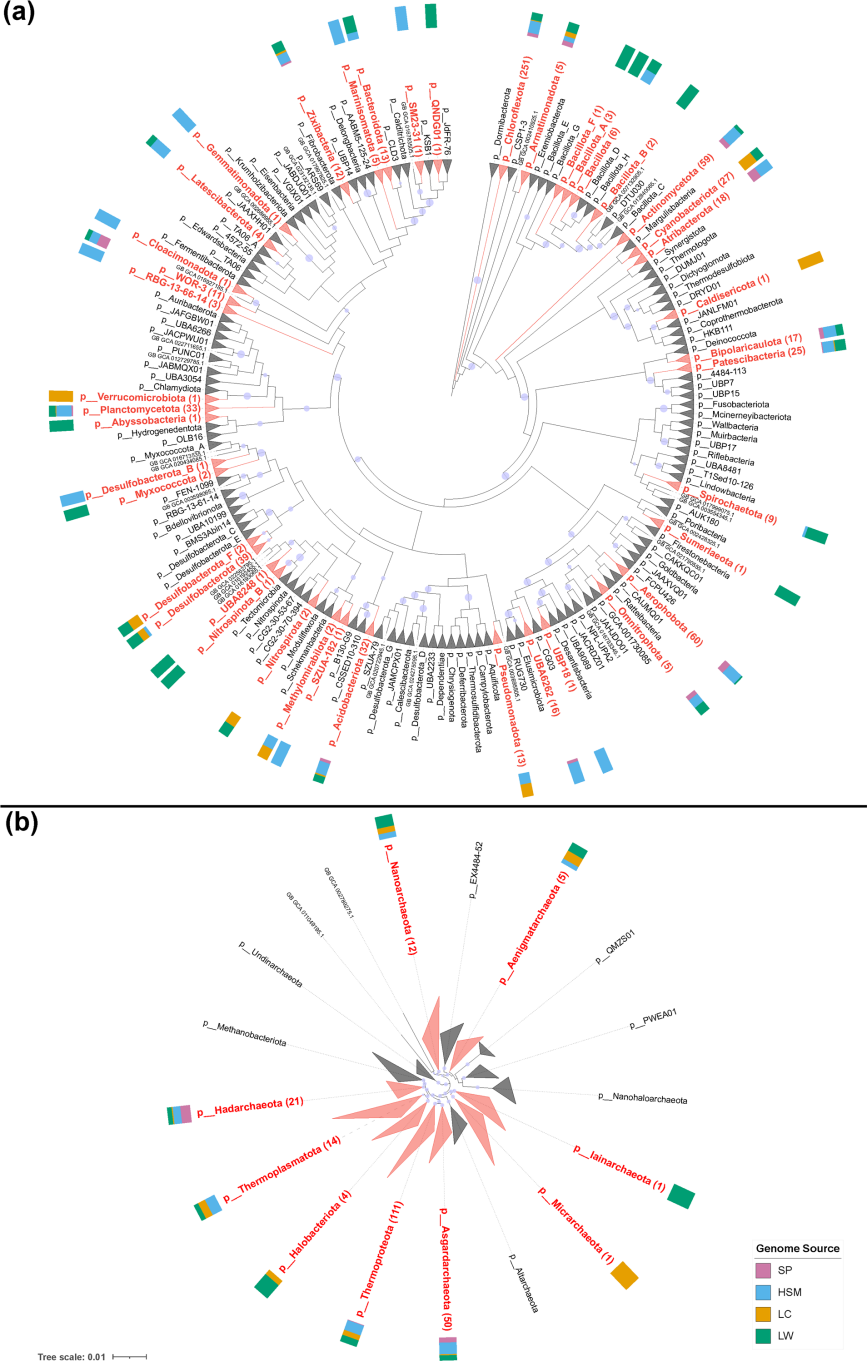
Phylogenetic tree of MAGs. (a) Bacterial MAGs. The alignment was based on a concatenated set of 120 protein markers (subsampled to a maximum of 42 sites each, resulting in a total alignment length of 5035 sites) from the 690 bacterial MAGs recovered in this study and 80 789 taxa. These taxa encompass bacterial species representatives from GTDB release 08-RS214. Maximum-likelihood analysis was performed using FastTree under the WAG+CAT model and optimized with Gamma20 likelihood. The tree was rooted on the Dormibacterota, and branch lengths were ignored. (**b)** Archaeal MAGs. The alignment was based on a concatenated set of 122 protein markers (subsampled to 42 sites each, resulting in a total alignment length of 5124 sites) from 2651 taxa. These taxa encompass archaeal species representatives from GTDB release 06-RS202. Maximum-likelihood analysis was performed using IQ-TREE with 1000 ultrafast bootstraps under the LG+C10+F+G+PMSF model. The tree is rooted on the Undinarchaeota.

Based on taxonomic assignment by GTDB-Tk [55], the recovered MAGs from coastal and deep subseafloor sediments represent 42 bacterial and 9 archaeal phyla (Figs4^ 5^ and S4b). Among the 686 bacterial MAGs, over one-third (251 MAGs) were identified as *Chloroflexota*, with the majority assigned to the class *Dehalococcoidia* (201 MAGs) and the rest to *Anaerolineae* (50 MAGs). The remaining bacterial MAGs represent a large variety of taxa, including the phyla of *Aerophobota* (60 MAGs), *Actinobacteriota* (57 MAGs), *Desulfobacterota* (39 MAGs) and *Planctomycetota* (33 MAGs) (Figs 5a and S4b). Most archaeal MAGs were classified as *Thermoproteota* (111 MAGs)*, Asgardarchaeota* (50 MAGs) and *Hadarchaeota* (21 MAGs) (Figs 5b and S4b). One MAG was assigned to *Cutibacterium acnes* (Table S7), a taxon that has been identified as a potential contaminant, and was subsequently excluded from the metabolic inference. Overall, the taxonomic distribution of recovered MAGs aligned well with the lineages detected in our community profiles (Fig. S4b). A *de novo* phylogenetic analysis revealed high taxonomic novelty among MAGs from established phyla, including one novel class-level MAG assigned to the phylum *Sumerlaeota*, 7 novel order-level MAGs in 6 classes, 58 novel family-level MAGs in 21 orders and 277 MAGs that are novel at the genus level (Table S7).

A total of 35 MAGs were assigned to 8 bacterial phyla that are only annotated with placeholder names in GTDB R214 (Table S8), comprising QNDG01 (1 MAG), RBG-13-66-14 (3 MAGs), SM23-31 (1 MAG), SZUA-182 (1 MAG), UBA6262 (16 MAGs), UBP18 (1 MAG), WOR-3 (11 MAGs) and UBA8248 (1 MAG). We recovered high-quality MAGs containing a full-length or near full-length 16S rRNA sequence (Table S8) for three of these phyla, namely, UBA8248, RBG-13-66-14 and UBA6262, all of which were resolved as monophyletic lineages in our phylogenomic inference (Fig. S8). Adhering to the quality criteria set by the SeqCode [79], a code of nomenclature under which genome sequences serve as nomenclatural types, we propose type material and higher ranks from family to phylum.

### Description of novel phyla and type material

#### *Tangaroaeota* phyl. nov. (RBG-13-66-14)

*Tangaroaeota* phyl. nov., named after Tangaroa, the god of the sea, lakes, rivers and creatures that live within them, in Māori mythology. The name was chosen to honour the sampling site of the type genome at the HSM in international waters off the coast of New Zealand. The nomenclatural proposals for this phylum name, the type genus from which the name was derived, the type species and for associated higher ranks, i.e. *Tangaroaeaceae* fam. nov., *Tangaroaeales* order nov. and *Tangaroaeia* class nov., are provided in (Table 1). The type species is *Tangaroaea hikurangi* sp. nov., named after the sampling site, the HSM in international waters off the coast of New Zealand. This uncultured species is represented by the type genome ‘HSM1520D_18H4_40’, whose genome size is 2.3 Mb, with the presence of 23S, 16S and 5S rRNA genes (Table S8). *T. hikurangi* is inferred to be organoheterotrophs that relies on chemical compounds such as glucose and fatty acids to conserve energy. The type genome neither carries genes for sulphur oxidation or sulphate reduction nor the reduction or oxidation of nitrogen compounds (Table S9). *Tangaroaeota* phyl. nov. has a global distribution across marine brackish and freshwater habitats and was mainly present in public shotgun metagenome datasets labelled as sediment metagenome (Fig. S8a), marine sediment metagenome and salt marsh metagenome, albeit the highest relative abundances were found in a hydrothermal vent metagenome from Guaymas Basin, Mexico.

**Table 1.** Proposed names for bacterial genera and associated higher ranks. Included are the designated MAGs for each type species. All names have been submitted for validation under the SeqCode, including the type species *Tangaroaea hikurangi* (https://seqco.de/i:49686), Ryujinia shimokita (https://seqco.de/i:49692), *Spongiamicus weybense* (https://seqco.de/i:49702)

GTDB taxonomy (parent taxa)	Proposed genus and species name	GTDB-pl	Type material							
			MAG ID & BioS	Com %	Con %	GC %	Genome size (Mb)	5S	16S bp	23S
**p__Tangaroaeota;c__Tangaroaeia**	Tangaroaea									
o__Tangaroaeales;f__Tangaroaeaceae;g__Tangaroaea	Tangaroaea hikurangi	RBG-13-66-14	HSM1520D_18 H4_40SAMN46525287	97.96	0.3	52.29	2.40	1	966	1
**p__Ryujiniota;c__Ryujiniia;**	Ryujinia									
o__Ryujiniales;f__Ryujiniaceae;g__Ryujinia	Ryujinia shimokita	UBA6262	SP_28 H5_5SAMD00885949	96.99	0.65	41.66	2.11	1	1557	1
**p__ Spongiamicota;c__ Spongiamicia;**	Spongiamicus									
o__ Spongiamicales;f__ Spongiamicaceae;g__ Spongiamicus	Spongiamicus weybense	UBA8248	LW2018_55 cm_44SAMN46305810	99.1	4.85	58.01	4.07	1	829	1

Com %estimated completeness in %Contestimated contamination in %Genome sizeestimated genome size in MbGTDB-plformer GTDB placeholder nameMAG ID & BioSMAG name of the designated type genome, and the NCBI BioSample ID5S5S rRNA count16S16S rRNA length in bp23S23S rRNA count

#### *Ryujiniota* phyl. nov. (UBA6262)

*Ryujiniota* phyl. nov., named after Ryūjin (龍神, lit. ‘Dragon God’) the protector deity of the sea in Japanese mythology, to honour the sampling site of the type genome in international waters off the coast of Japan. An overview of the nomenclatural proposals for this phylum name, the type genus from which the name was derived, the type species and for associated higher ranks, i.e. *Ryujiniaceae* fam. nov., *Ryujiniales* order nov. and *Ryujiniia* class. nov., are provided in Table 1. The type species is *Ryujinia shimokita* sp. nov., named after the sampling site, the international waters off the SP in Japan. This uncultured species is represented by the genome ‘SP_28H5_5’, whose genome size is 2.0 MB, with the presence of 23S, 16S and 5S rRNA genes (Table S8). *R. shimokita* is inferred to have a chemolithotrophic lifestyle, since this species encodes a nearly complete Wood–Ljungdahl (WL) pathway. It also has the potential for a heterotrophic lifestyle with substrate-level phosphorylation through glycolysis, while it lacks most genes for the citrate cycle and beta-oxidation. The type genome does not encode the ability for the oxidoreduction of nitrogen or sulphur compounds (Table S9). *Ryujiniota* phyl. nov. has a global distribution and was mainly present in public shotgun metagenome datasets labelled as marine sediment metagenome (Fig. S8b), peat metagenome, sediment metagenome, soil metagenome and groundwater metagenome. The highest relative abundances of *Ryujiniota* were found in Costa Rica margin subsurface and east pacific subsurface metagenomes with 15.75% and 15.33%, respectively (additional data).

#### *Spongiamicota* phyl. nov. (UBA8248)

*Spongiamicota* phyl. nov., named after sponge, the colloquial term for the eukaryotic lineage porifera, and after amicus, the Latin term for friend, to honour the fact that this lineage shows their highest relative abundances in sponge metagenomes. A summary of nomenclatural proposals for this phylum name, the type genus from which the name was derived, the type species and for associated higher ranks, i.e. *Spongiamicaceae* fam. nov., *Spongiamicales* order nov. and *Spongiamicia* class. nov., are provided in Table 1. The type species is *Spongiamicus weybense* sp. nov., named after the sampling site of the type genome, LW, a saltwater lake in Queensland, Australia. This uncultured species is represented by the genome ‘LW2018_55cm_44’, whose genome size is 4.0 MB, with the presence of 23S, 16S and 5S rRNA genes (Table S8). The highest relative abundances of this phylum were observed in sponge metagenomes from the marine sponge *Aplysina aerophoba* from the Gulf of Piran, Slovenia, and sperm DNA of *Galaxea fascicularis* from an undisclosed coral reef sample. *S. weybense* is inferred as organoheterotrophs but could potentially fix CO_2_ via the WL pathway. It also encodes genes for nitrogen fixation (Table S9). *Spongiamicota* phyl. nov. has a global distribution and was mainly present in public shotgun metagenome datasets labelled as marine metagenomes, marine sediment metagenomes, seawater metagenome and sponge metagenome (Fig. S8c).

## Inferred core functions of microbial communities and potential key players in nutrient cycling

**Fig. 6.**
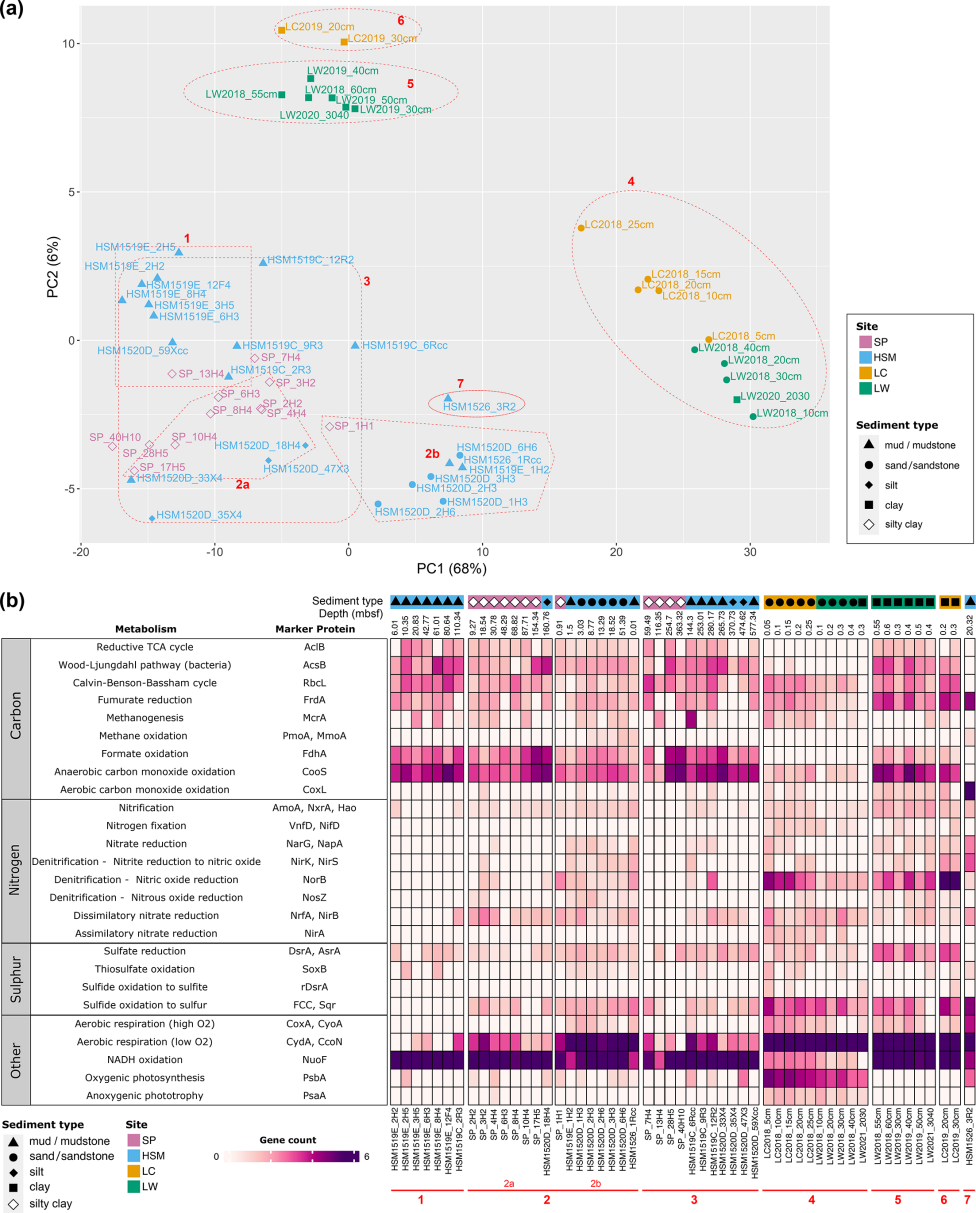
Inferred functional profiles of microbial communities. (a) Principal component analysis of functional profiles. Encoded genes from metagenomic assemblies were screened by prodigal and annotated with KOfamscan. The gene count values were then transformed with DESeq2 in R. (**b)** Metabolic capabilities of microbial communities. A list of encoded marker proteins was selected based on Fig. 2 in Chen *et al*. [112]. For reactions involving multiple encoded marker proteins, counts were summed with the exceptions of nitrification marker genes (*hao* and *narG*) and nitrogen fixation genes (*vnfD* and *nifD*), which were averaged as both genes are required for functioning. Samples are ordered by sampling sites and sediment depths. Methanogenetic McrA and sulphate-reducing DsrA genes were identified through phylogenetic analysis (shown in Figs S9 and S10 for McrA and DsrA, respectively). Normalized gene counts (square root transformed) used to create this figure can be found in Table S12. The marker protein KOs are aclB (K15231), acsB (K14138), rbcL (K01601), frdA (K00244), mcrA (K00399), pmoA (K10944), fdhA (K22516), cooS (K00198) and coxL (K03520).

The comparative analysis of encoded core metabolic functions across all samples (Table S10) revealed a rich repertoire of genes for biogeochemical cycling, including carbon fixation, nitrate and ammonia cycling and sulphate metabolism. However, whether these inferred functions are encoded by cellular entities (dead or alive) or by extracellular DNA (also known as relic DNA) remains to be determined. Thus, for the purpose of this article, we assess the metabolic potential of the total DNA pool that was present in our samples. We recovered three main functional groups, which represented metagenomes from (1) the shallowest, sandy layers of coastal sediment, containing all samples of the taxonomic cluster 4, established by the Euclidean distance-based beta diversity analysis (Fig. 3a), then (2) the deeper clay-dominated layers of coastal sediments, containing the taxonomic clusters 5 and 6 and from (3) all deep subseafloor sediments (Fig. 6a). Within the third group, the functional patterns also followed the taxonomic clusters, although there was considerable overlap between clusters (Fig. 6a). Next, we assessed the main differences between these functional groups, based on marker protein analysis (Fig. 6b). The functional group comprising cluster 4, i.e. samples form sandy coastal sediments, showed an elevated potential for oxygenic photosynthesis, but also for denitrification (nitric oxide reduction), and was the only group exhibiting limited NADH oxidation abilities and the ability for anoxygenic phototrophy. The group of coastal clay samples, including clusters 5 and 6, was characterized by higher proportions of genes encoding enzymes involved in carbon cycling, including carbon fixation through the reductive tricarboxylic acid (TCA) cycle (AclB) and the bacterial WL pathway (AcsB), as well as the anaerobic carbon monoxide oxidation (CooS), anaerobic fumarate reduction (FrdA) and NADH oxidation (NuoF), compared with cluster 4 (Fig. 6b). Furthermore, aerobic respiration under low oxygen conditions was as prevalent, and sulphate reduction (DsrA and AstA) and nitric oxide reduction (NorB) genes were enriched compared with most other clusters (Fig. 6b). The third functional group, containing the deep subseafloor samples from the taxonomic clusters 1, 2 and 3, were characterized by formate and anaerobic carbon monoxide oxidation as well as NADH oxidation (Fig. 6b). Cluster 2b differed in that formate and anaerobic carbon monoxide oxidation were less abundant, and by showing the highest potential for aerobic respiration under low oxygen conditions. These results align with the shallow depths of the samples in this cluster, which were, with one exception, all recovered from 0.01 to 18.52 mbsf. Cluster 7, consisting of only one deep subseafloor sample from 20.3 mbsf, proved to also be an outlier based on core functions, showing the highest ability for aerobic carbon monoxide oxidation and fumarate, nitrate and nitrite reduction (Fig. 6b).

To identify the taxa encoding these functions, we compared the presence of our marker proteins across all MAGs (Table S11, Data S1). The most widespread inferred inorganic carbon fixation pathways were anaerobic carbon monoxide oxidation, represented by CooS, and the bacterial WL pathway (AcsB), which were present in 29 and 23 phyla, respectively, including the newly proposed phyla *Ryujiniota* phyl. nov. (UBA6262) and *Spongiamicota* phyl. nov. (UBA8248) (Table S11a), although the possibility that these carbon fixation pathways are used in reverse cannot be ruled out. An exception to this pattern was MAGs assigned to the archaeal phylum *Thermoproteota* and the bacterial phyla *Hadarchaeota*, *Iainarchaeota*, *Cyanobacteriota* and *Pseudomonadota*, which almost exclusively encoded the key enzyme (RbcL) of the Calvin–Benson cycle. *Thermoproteota* and *Halobacteriaota* were the only phyla encoding methane oxidation and methanogenesis markers (McrA), the latter were confirmed by phylogenetic inferences (Fig. S9). Heterotrophic carbon utilization by formate oxidation was detected in 16 phyla, including the dominant taxa *Chloroflexota*, *Aerophobota* and *Atribacterota* but also *Ryujiniota* phyl. nov. (UBA6262). Fumarate reduction was not as common and mainly restricted to *Desulfobacterota*, *Chloroflexota* and *Aerophobota* (Table S11a). A noteworthy case was the phylum *Desulfobacterota*_B (former name *Binatota*), which encoded only aerobic carbon monoxide oxidation (coxL) as the sole carbon cycling marker protein. The 3-hydroxypropionate bicycle, a carbon fixation pathway reported for several members of the phylum *Chloroflexota* [80], in particular in the class *Chloroflexaceae* [81], was only partially encoded across all samples. No key genes encoding 3-hydroxypropionate dehydrogenase, succinate semialdehyde, 4-hydroxybutanoate:CoA ligase and succinyl-CoA reductase were detected in any of the samples, suggesting that microbial communities in the coastal and deep-sea subseafloor of the Western Pacific Region do not rely on 3HP/4HB or DC/4HB metabolism for carbon fixation.

In nitrogen cycling, nitrate reduction genes were most common and showed higher relative abundances in shallow coastal compared with deep-sea sediments (Fig. 6b, Table S11b). Complete reduction of nitrate to ammonia via the dissimilatory nitrate reduction pathway was encoded by *Mycobacteriales* (*Actinobacteriota*) and *Burkholderiales* (*Pseudomonadota*), which were abundant community members in coastal sediments (Table S3). Ammonia produced by nitrogenous organic compound degradation, or resulting from dissimilatory nitrate reduction, could be utilized by members of the archaeal order *Nitrososphaerales*, which encoded ammonia monooxygenase (AmoA). At coastal sites, key denitrification genes were detected in the orders 4572-78 (*Chloroflexota*) and *Rhizobiale* and *Burkholderiales* (*Pseudomonadota*). Nitrogen produced by chemical and biological processes, including denitrification, could be fixed by another *Burkholderiales* taxon, the family *Sulfuricellaceae*, which encoded the nitrogenase molybdenum-iron protein (NifD). Nitrogen fixation and nitrate reduction and denitrification are likely vital components of the nitrogen cycle in the marine sediments analysed in this study.

The majority of metagenomes in our dataset encoded sulphide oxidation pathways (Fig. 6b, Table S11c), highlighting the importance of the inferred sulphur cycling among microbial communities in coastal and deep-sea sediments. Genes for sulphide dehydrogenase (FCC) and sulphide:quinone oxidoreductase (Sqr), which are enzymes that catalyse the oxidation of hydrogen sulphide to elemental sulphur, showed higher relative abundances in LC and LW and were the least common among HSM mud sediments. Dissimilatory sulphate reduction, inferred based on dissimilatory sulphite reductase (DsrAB) and adenylyl sulphate reductase (AprAB) genes, was detected across most samples, which is expected given the high proportions of sulphate-reducing bacteria, such as *Desulfobacterales* and *Desulfobulbales*, in our dataset (Table S3). Since DsrAB can also be reverse operating, i.e. acting oxidatively (rDsrAB), we conducted a phylogenetic analysis to further discriminate between both reactions. While most DsrA sequences were identified as reductive, we found that nine from LC metagenomes clustered with the oxidative group (Fig. S10). Contigs containing these LC rDsrA genes were binned into six *Burkholderiales* genomes, which also contained ATP sulfurylase (Sat), Apr and FCC genes (Table S11c), further supporting a role in hydrogen sulphide oxidation and sulphate production of this taxon [82]. Sulphate could also be generated through thiosulphate oxidation, which is supported by the presence of a complete SOX complex in HSM, LC and LW metagenomes (Table S11c). MAGs containing SoxB genes were assigned to the *Pseudomonadota* (former *Proteobacteria*) orders *Rhizobiales*, *Burkholderiales* and HK1 (Table S11c).

## Discussion

Our metagenomic study explored patterns in diversity, taxonomic profiles and inferred functional capabilities to better understand microbial community distributions in shallow coastal and deep subseafloor sediments in the Western Pacific Region. Alpha diversity assessments revealed significantly higher diversities in shallow coastal compared with deep-sea sediments, which is likely driven by the greater availability of organic compounds, nutrients and terminal electron acceptors, increased disturbance by hydrodynamic forces and lower community stability imposed by temperature changes [4,83, 84]. Beta diversity revealed taxonomic clusters, which were mostly attributed to sediment type (Fig. 3a, b), although chemico-physical parameters, including depth below seafloor, pH and sulphate levels, were also inferred to impact microbial communities (Fig. 3c). These factors have been reported previously as important forces shaping sediment microbial compositions [4,7], whereby the diverse physical properties in deep subseafloor sediments have been linked to the presence of different levels of organic compounds during sediment composition and subsequent diagenesis [85]. Our results suggest that, overall, these processes can generate similar conditions and therefore similar microbial communities across the Western Pacific Region. For example, the taxonomic clusters 2 and 3 in our analysis contain both subsurface sediment samples from SP and HSM, two sites that are over 8500 km apart. However, we also found exceptions to this pattern. For example, the community profile of the 20.3 mbsf HSM sample, the only deeper sample recovered from site U1526, was unique compared with all other samples in our dataset (Fig. 4). Sediments at this site differ from our other HSM sites, since U1526 is thought to consist of subaerial eruption products that have been reworked by wave action [86]. The unique microbial community of the 20.3 mbsf sample, which is also an outlier based on core functions (Fig. 6b), supports previous findings that local geological variables have a strong influence on community compositions [87].

Investigating the lineages making up the taxonomic clusters, in combination with an analysis of inferred functional groupings, provided insights into possible ecological roles of microbial communities in shallow coastal and deep-subsurface sediments. The functional grouping, consisting of cluster 4 (Fig. 6b), i.e. sand-type lake sediments from LW and LC collected from 0.1 to 0.4 mbsf, suggests that this habitat enables the microbial community to utilize oxygen and/or sunlight. This conclusion is supported by the detected genes for aerobic respiration (e.g. CoxA and CyoA) and photosynthesis (e.g. PsaA and PsbA). We recovered MAGs for the four *Cyanobacteriota* families *Cyanobacteriaceae*, *Microcystaceae*, *Xenococcaceae* and *Phormidesmiaceae* from this shallow coastal sediment and found that these taxa are present with relative abundances up to 4.8% (Table S3). Three of the four families (*Cyanobacteriaceae*, *Microcystaceae* and *Xenococcaceae*) have members known to thrive under low light and marginal oxygen conditions [88,90]. However, light and oxygen availabilities are likely temporal since increased wind and wave activities have been reported to resuspend sediments in these marine lakes [91], following seasonal patterns [92]. Interestingly, one LW sample collected as ‘clay sediment’ (LW2020_2030) was also grouped into this cluster, consisting otherwise of sand samples. This sample came from the start of the clay layer at 0.2 mbsf, at the interface of clay and sand, and likely resembled conditions closer to those in sandy sediments. We did not detect *Cyanobacteria* in this LW sample; however, genes for oxygenic photosynthesis (e.g. PsbA) and low oxygen aerobic respiration (e.g. CydA) were present, supporting the observed functional clustering. The recovery of MAGs was unsuccessful for this particular sample, and therefore, a future deep-sequencing effort is warranted to explore the microbial players inhabiting the interface between sandy and clay sediment layers in these coastal sediments.

The next functional grouping was also united by sediment type and consisted of the two clusters 5 and 6 (Fig. 6a), both representing microbial communities from coastal clay samples. In LW and LC, the clay layers are part of the deeper anoxic sediments, located underneath the sand deposits. Since the oxygen levels in the LW clay were below 0.2%, we conclude that these samples, collected from depths between ~0.3 and 0.6 mbsf, represent oxygen-limited or oxygen-depleted habitats. In oxygen-depleted sediments, nitrate reduction and denitrification are expected to be of importance [23]. Indeed, genes coding for reductases of nitrate (Nap and Nar), nitrite (Nir), nitric oxide (Nor) and nitrous oxide (Nos) were detected in MAGs from several taxa (Table S11), supporting the assumption that nitrate functions as important terminal electron acceptor [93] in these sediments. We measured a considerable amount of total Kjeldahl nitrogen, i.e. the sum of organic nitrogen compounds and ammonia, in the clay sediments of LW (480 mg kg^−1^) and LC (210 mg kg^−1^). The detected nitrogen pool might be supported by marine and terrestrial sources [94], but possibly also nitrogen fixation that could be carried out by members of *Cyanobacteriales* and *Burkholderiales*, since both lineages are known to fix nitrogen in soil/sediment [95,98] and MAGs of both lineages encoded nitrogenase genes (nirDKH). Inorganic nitrogen compounds such as nitrite and nitrate were not detected in the lake clay samples, nor was ammonia [37]. This suggests, together with the elevated Kjeldahl nitrogen measurements, that the bulk of nitrogen is present as organic compounds, indicating a fast turnover of bioavailable nitrogen in these areas [84,99].

The third functional group included all microbial communities from subseafloor sediments, although further separation into clusters 1, 2a and 2b could be observed (Fig. 6a). The limited availability of chemical-physical metadata for our subseafloor sediments restricted the inference of habitat-specific ecological roles of key microbial taxa. However, microbial communities in deep-subsurface sediments have been reported to play an important role in global biogeochemical cycling [100]. Our results support these findings and suggest that HSM microbes have the potential to be involved in nitrogen cycling. In HSM sediments, ammonia was measured in seven of the sequenced samples obtained from 3.03 to 265.7 mbsf. Ammonia concentrations increased with depth, and this increment was correlated (*r*=0.896, *P*-value=0.006) with the increasing abundance of genes involved in the reduction of nitrite to ammonia (NrfA and NirB), when excluding one outlier (Fig. S11). Ammonia is likely also supplied via the degradation of nitrogenous compounds such as aa degradation and urea degradation. However, the *ureABC* genes encoding the urease subunits alpha, beta and gamma that are involved in the catalysis of urea to ammonia were absent in most subseafloor samples (Table S10). Overall, the bulk of the resulting ammonia is likely used for aa biosynthesis. Ammonia monooxygenase (Amo), considered to represent the rate-limiting enzyme in nitrification by catalysing the oxidation of ammonia to hydroxylamine [101], was only present in low abundances in HSM sediments and seemed to be restricted to the order *Nitrososphaerales*. Our conclusion is that ammonia, produced by nitrogen fixation, nitrite reduction and possibly also nitrogenous compound degradation, could be utilized for aa biosynthesis via glutamine synthetase.

An involvement in sulphur cycling is also very likely, in deep-subsurface as well as shallow coastal sediments. In general, sulphur is a known key element in marine sediments due to its ability to act as an electron acceptor, carrier and donor. In particular, the oxidized form of sulphur – sulphate – is commonly the main electron acceptor for respiration in anoxic marine sediments [102]. We found this to be a unifying feature across deep-subsurface and shallow coastal sediments, since we detected genes for key catalytic enzymes involved in sulphur cycling in both habitats, including dissimilatory sulphite reductase (Dsr), S-sulfosulfanyl-l-cysteine sulfohydrolase (SoxB) and sulphide:quinone oxidoreductase (Sqr). In the shallow coastal clay layers, sulphide concentrations were significantly higher than those of sulphate (*P=0.031*). Thus, sulphate reduction might be the more common reaction, and could be a main source for elevated sulphate levels in these coastal sediments. This reaction could be driven by *Desulfobacterota* and *Chloroflexota*, as suggested by their higher relative abundances and the prevalence of inferred sulphate reduction genes (e.g. *dsrA*), detected in the MAGs from both phyla. At the same time, fermentation products such as formate, hydrogen or acetate may serve as electron donors. Biological oxidation of reduced sulphur compounds was inferred to be restricted to the upper layers of shallow coastal sediments, predominantly by class *Cyanobacteriia* in the sandy surface sediment layers of LC and LW and *Bacteroidia* in the deeper layers of these coastal lake sediments (>40 cm) and the HSM upper sediment layers (18.5 to 160.8 mbsf). These micro-organisms are inferred to use Sqr to oxidase sulphide and generate elemental sulphur and polysulphide. The oxidation reaction is usually coupled with electron acceptors with sufficient redox potentials [103] and likely linked to nitrate reduction in these habitats.

A limitation of our study is that the metabolic reconstruction based on inferences of metagenomic data can only provide limited insights into the directionality of the inferred reactions and pathways. For some reactions, such as sulphate reduction, gene phylogenies could resolve the direction (Fig. S10), whereas in other cases, the concentrations of molecules on either side of the equation can provide insights. For example, the region containing the HSM site was estimated to release considerable amounts of CO_2_ and N_2_, produced under increasing pressure and temperature from the subducted plate [104,105]. This overabundance of CO_2_ and N_2_ suggested that our inferred carbon and nitrogen fixation pathways are thermodynamically favourable reactions under these conditions. However, further studies, involving metabolic flux analysis, stable isotope labelling and possibly also metatranscriptomics and metabolomics, will be necessary to unambiguously clarify the directionality of microbial reactions in the examined sediments.

## Conclusion

Our metagenomic survey assessed community structures, identified functional clusters and recovered genomes from well-characterized and novel lineages that were inferred to contribute to nutrient cycling in shallow coastal and deep-subsurface sediments in the West Pacific Region. This study also highlighted the fact that benthic prokaryotic communities, especially marine deep-subsurface sediments, are a treasure trough for the recovery and description of novel prokaryotic lineages. Our phylogenetic, taxonomic and metabolic inferences will likely provide the foundation for future *in situ* assessments of metabolic activities targeting key lineages, a task that is especially challenging for deep-subsurface microbes that are difficult to access. Detailed, taxon-specific analyses are urgently needed for deep-subsurface habitats; e.g. radiotracer experiments have concluded that active methanogenic and sulphate-reducing populations exist in marine subsurface sediments [106]; however, the key microbial taxa and their metabolic pathways have remained largely unexplored. A promising approach to evaluate the activities of deep-subsurface microbes and to assess microbial interactions at the same time is a combination of enrichment cultures and meta-omic analyses, such as metatranscriptomics, proteomics and metabolomics. This approach has been recently employed to study sediment microbes that were previously referred to as viable but non-culturable [107]. Admittedly, obtaining enrichments and axenic cultures of deep biosphere microbes comes with its inherent challenges such as fine-tuning nutrient concentrations, temperature and hydrostatic pressure. Although this task is achievable, since several piezophilic deep-sea species have been cultured over the past 2 years [108]. Next to revealing taxonomic novelty, and improving our understanding of ecosystem functions, exploring cryptic deep-subsurface taxa can also open up applied avenues, such as the discovery of novel, bioactive secondary metabolites with antimicrobial and anticancer activities [109].

## supplementary material

10.1099/mgen.0.001351 Text S1.

10.1099/mgen.0.001351 Supplementary figures.

10.1099/mgen.0.001351 Table S1.

## Abbreviations

DV: drilling vessel
GTDB: Genome Taxonomy Database
HSM: Hikurangi Subduction Margin
IODP: International Ocean Discovery Program
LC: Lake Cootharaba
LW: Lake Weyba
MAGs: metagenome-assembled genomes
mbsf: metres below the seafloor
mOTUs: marker gene-based operational taxonomic units
PERMANOVA: permutational multivariate ANOVA
SMTZ: sulphate–methane transition zone
SP: Shimokita Peninsula
WL: Wood–Ljungdahl
WRC: whole round core
