# Supplementary material for: Metagenomic insights into taxonomic and functional patterns in shallow coastal and deep subseafloor sediments in the Western Pacific
Source: Microb Genom. 2025 Mar 18;11(3):001351. doi: 10.1099/mgen.0.001351 (PMC11920076; doi:10.1099/mgen.0.001351)
Supplement: Text S1. [file mgen-11-01351-s001.pdf]

Suppl. Text

Additional results

Sedimentology. The deep subseafloor cores from the three HSM sites included a wide range of sediment types (**Figure S1a**), as described in the Proceedings of the International Ocean Discovery Program (IODP) (1). In brief, site U1519 and U1526 samples analysed in this study were characterised by mud, at the sampled depths of 1.5 to 280.2 mbsf, and 0.01 to 20.3 mbsf, respectively. For site U1520, samples of sandstone and silt sediments dominated from 3 to around 500 mbsf, followed by calcareous mudstone at 577 mbsf (**Figure S1a**). Deep subseafloor sediments from SP at site C0020, spanning depths from 0.9 - 363.3 mbsf, consisted primarily of diatom-rich silty clay (2,3). Sediments from the shallow coastal saline lakes LC and LW, were characterised by yellow, loose sand at the surface that gradually became more compacted and turned black at around 10 cm, followed by dense clay at about 20 - 40 cm depth, with slight variations between cores (**Figure S1b**).

Geochemistry. We analysed a subset of geochemistry profiles from the HSM and SP sites (4–7), by focusing on data most relevant for microbial community distributions. The deep subsurface SP site C0020 was characterised by a shallow sulfate–methane transition zone (SMTZ) at around 4 mbsf, and by the presence of methane across all sampled depths below the SMTZ, as well as low methane concentrations above this zone, as described previously (2). Sulfate did not decrease with depth and remained at low levels from 5 to 363 mbsf (0.02 - 2.78 mM) (2), suggesting that sulfate reduction and methanogenesis might coexist in these sediments (8). In addition, alkalinity and ammonia concentrations were reported to significantly correlate with increasing depth (2).

Two deep subseafloor sites at HSM were characterized by a rapid decrease in sulfate to depletion at the SMTZ at about 8 and 28 mbsf, at site U1519 and U1520, respectively (**Figure S1b-c**). Below the SMTZ, methane was detected to a depth of 144 and 464 mbsf, at site 1519 and 1520 respectively. At site U1520, at about 480 mbsf sulfate increased again to surface levels, whereas phosphate decreased towards depletion, and alkalinity decreased to average seawater values. In the shallower core samples (0.01 to 20.3mbsf) from site U1526, sulfate levels remained constant (around 28 mM), and no methane was detected, suggesting that the SMTZ at this site was below the sampled depths (**Figure S1d**). Across our analysed samples from site U1519 and U1520, we found several correlations between the geochemical sediment data (**Figure S12, Table S13**). Calcium was negatively correlated with alkalinity levels at site U1519 ($r = -0.85$ for U1519), and magnesium levels were decreasing with depth at both sites ($r = -0.72$ for U1519; $r = -0.99$ for U1520). Additionally, sulfate and total nitrogen concentrations correlated significantly ($r = 0.94$ for U1519; $r = 0.83$ for U1520), and so did the concentrations of organic carbon and nitrogen (**Table S13a**).

For the coastal sediments in LW and LC, chemistry data were only available for the clay layers, observed deeper than 0.2 mbsf. Oxygen levels below 0.2%, at the detection limit of our method, were measured in dense clay samples at LW (core LW2020), suggesting that these layers were below the transition from aerobic to anaerobic conditions. Sediments from both lakes were also characterised by the presence of sulfate, with LW showing up to 20 times higher concentrations than LC (**Table S1**). Phosphate and nitrate levels were also up to 2.7 and 1.6 times higher in LW, compared to LC. In addition, LW sediments were more alkaline with an average pH of 8.53, compared to the average pH of 7.1±0.2 at LC, and also in

comparison to the average pH of all HSM sediments of 7.8±0.1. Significant correlations ($r > 0.9$) were found between concentrations of sulfide, sulfur, phosphorus, and total organic carbon, across the analysed samples for LW (**Table S13b**). No correlations among the geochemical sediment data could be calculated for LC sediments due to the limited number of samples with metadata (**Table S1**).

References

1. al DMS et. Expedition 372B/375 summary [Internet]. [cited 2024 Jun 27]. Available from: http://publications.iodp.org/proceedings/372B_375/101/372B375_101.html

2. Nunoura T, Takaki Y, Shimamura S, Kakuta J, Kazama H, Hirai M, et al. Variance and potential niche separation of microbial communities in subseafloor sediments off Shimokita Peninsula, Japan. Environmental Microbiology. 2016;18(6):1889–906.

3. Tanikawa W, Tadai O, Morono Y, Hinrichs KU, Inagaki F. Geophysical constraints on microbial biomass in subseafloor sediments and coal seams down to 2.5 km off Shimokita Peninsula, Japan. Progress in Earth and Planetary Science. 2018 Sep 26;5(1):58.

4. Inagaki F, Hinrichs KU, Kubo Y, Bowles MW, Heuer VB, Hong WL, et al. Exploring deep microbial life in coal-bearing sediment down to ~2.5 km below the ocean floor. Science. 2015 Jul 24;349(6246):420–4.

5. Wallace, L.M., Saffer, D.M., Barnes, P.M., Pecher, I.A., Petronotis, K.E., LeVay, L.J., and the Expedition 372/375 Scientists. Site U1520 [Internet]. Available from: http://publications.iodp.org/proceedings/372B_375/105/372B375_105.html

6. Wallace, L.M., Saffer, D.M., Barnes, P.M., Pecher, I.A., Petronotis, K.E., LeVay, L.J.,, and the Expedition 372/375 Scientists. Site U1526 [Internet]. Available from: https://doi.org/10.14379/iodp.proc.372B375.106.2019

7. Wallace, L.M., Saffer, D.M., Barnes, P.M., Pecher, I.A., Petronotis, K.E., LeVay, L.J.,, and the Expedition 372/375 Scientists. Site U1519 [Internet]. Available from: https://doi.org/10.14379/iodp.proc.372B375.104.2019

8. Sela-Adler M, Ronen Z, Herut B, Antler G, Vigderovich H, Eckert W, et al. Co-existence of Methanogenesis and Sulfate Reduction with Common Substrates in Sulfate-Rich Estuarine Sediments. Front Microbiol [Internet]. 2017 May 5 [cited 2024 Mar 20];8. Available from: https://www.frontiersin.org/journals/microbiology/articles/10.3389/fmicb.2017.00766/full
