# Supplementary material for: Metagenomic insights into taxonomic and functional patterns in shallow coastal and deep subseafloor sediments in the Western Pacific
Source: Microb Genom. 2025 Mar 18;11(3):001351. doi: 10.1099/mgen.0.001351 (PMC11920076; doi:10.1099/mgen.0.001351)
Supplement: Supplementary figures. [file mgen-11-01351-s002.pdf]

Supplementary figures

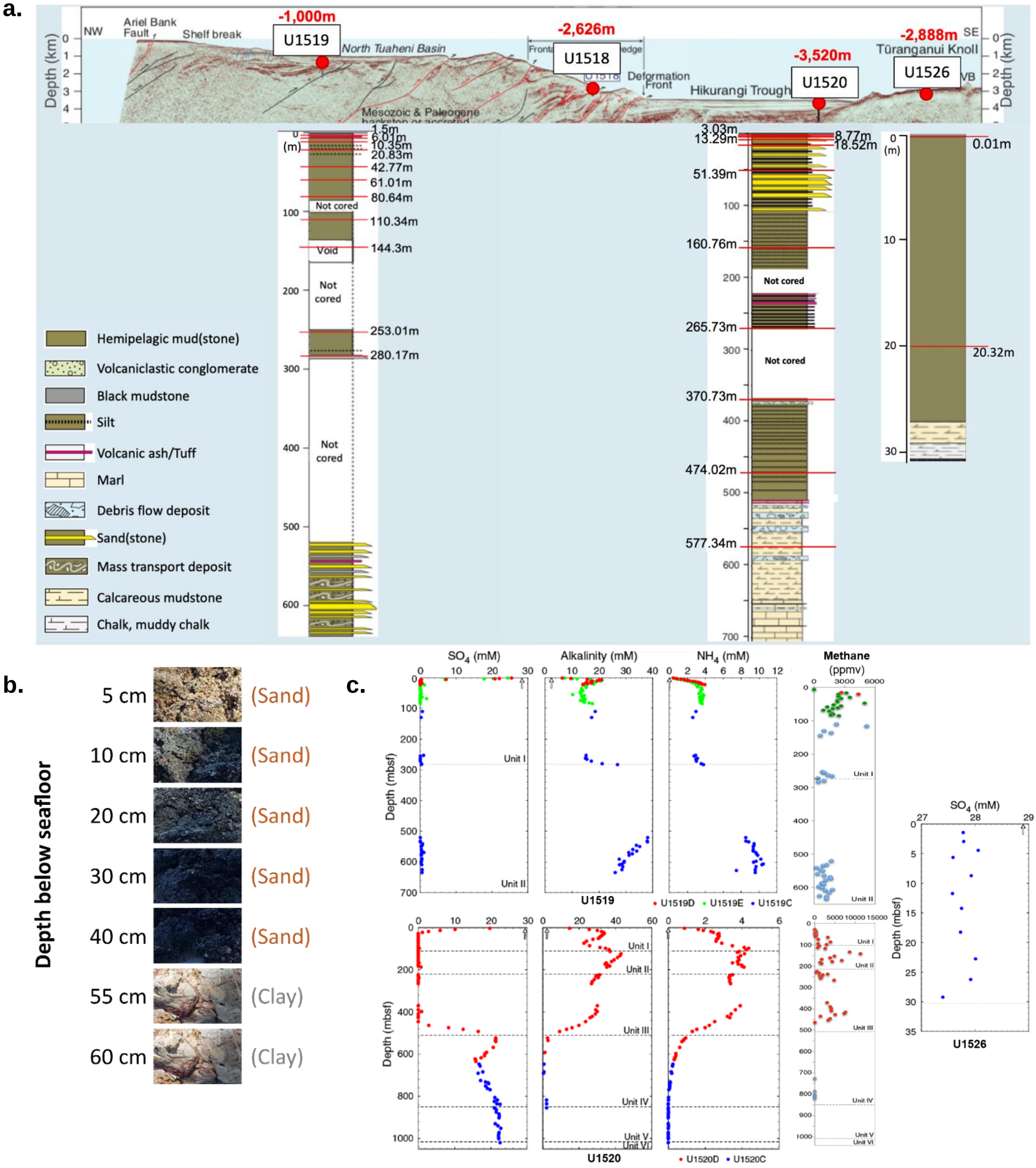

Figure S1 | Sediment profiles. a. Sediment profile of Hikurangi Subduction Margin. The figure was modified from Saffer et al. Samples analysed in this study were marked with red horizontal lines with depths labelled aside. Water depths were indicated with red labels on top of each site. **b.** Sediment profile of Lake Weyba (core 2018). Shown is a Lake Weyba (LW) core from 2018 characterised by loose sand at the surface that became compacted and turned black at around 10 cm, followed by dense clay layers at about 20 - 40 cm depth. Note that the 2019 and 2020 LW cores (not pictured) had a slight variation of these layers in terms of depth, i.e. the clay layers started shallower at around 20 - 30cm in 2019, and 10-20cm in 2020. **c.** Geochemistry of HSM

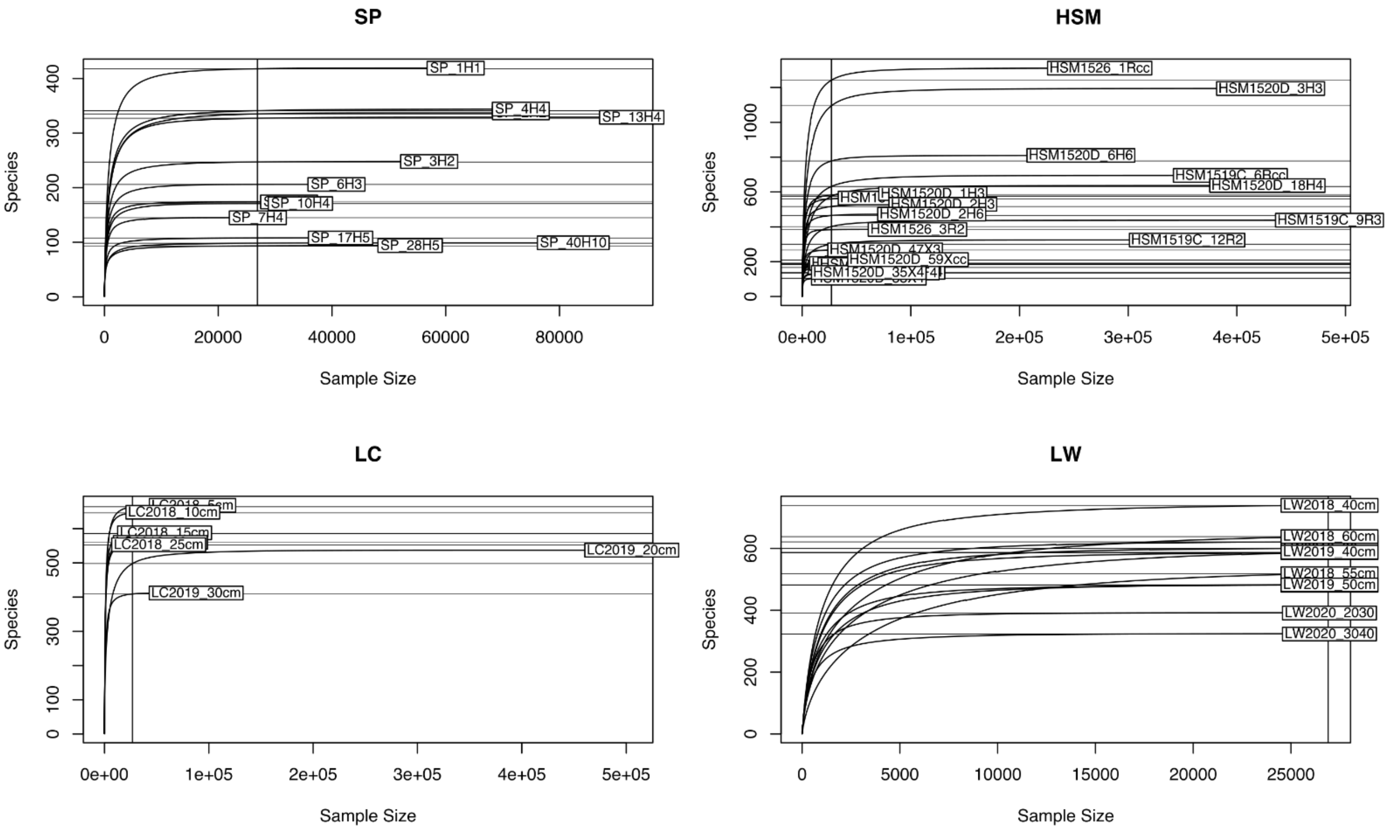

Figure S2 | Rarefaction curves of microbial communities at different sampling locations.

a. **b.** **c.**

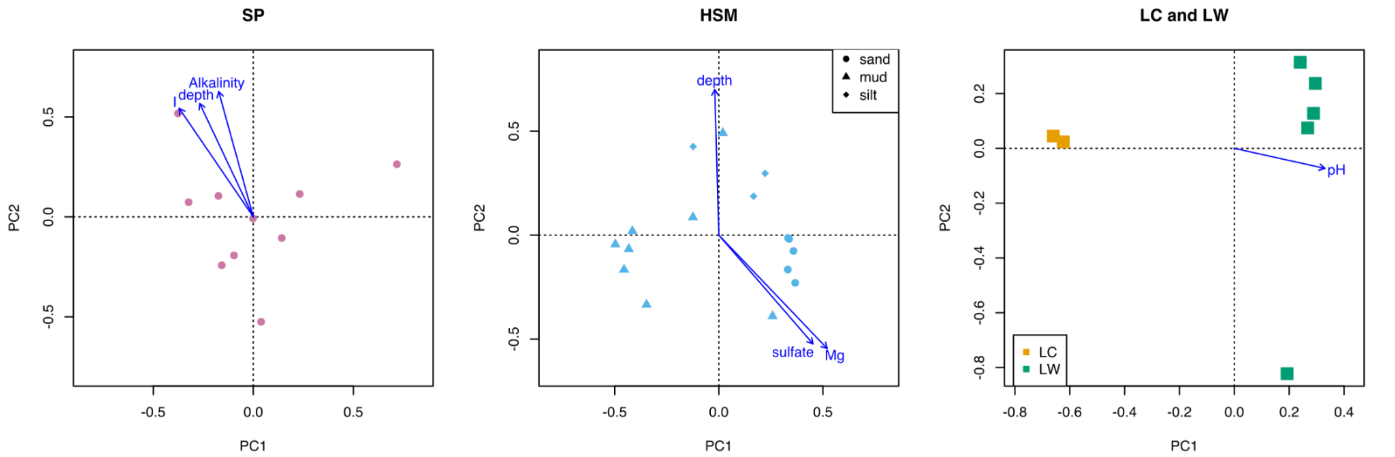

Figure S3 | Variance-based principal component analysis, fitted with environmental factors selected from a stepwise PERMANOVA. a. Principal component analysis on Shimokita Peninsula samples (SP). **b.** Principal component analysis on Hikurangi Subduction Margin (HSM) samples. **c.** Principal component analysis on Lake Cootharaba (LC) and Lake Weyba (LW) samples.

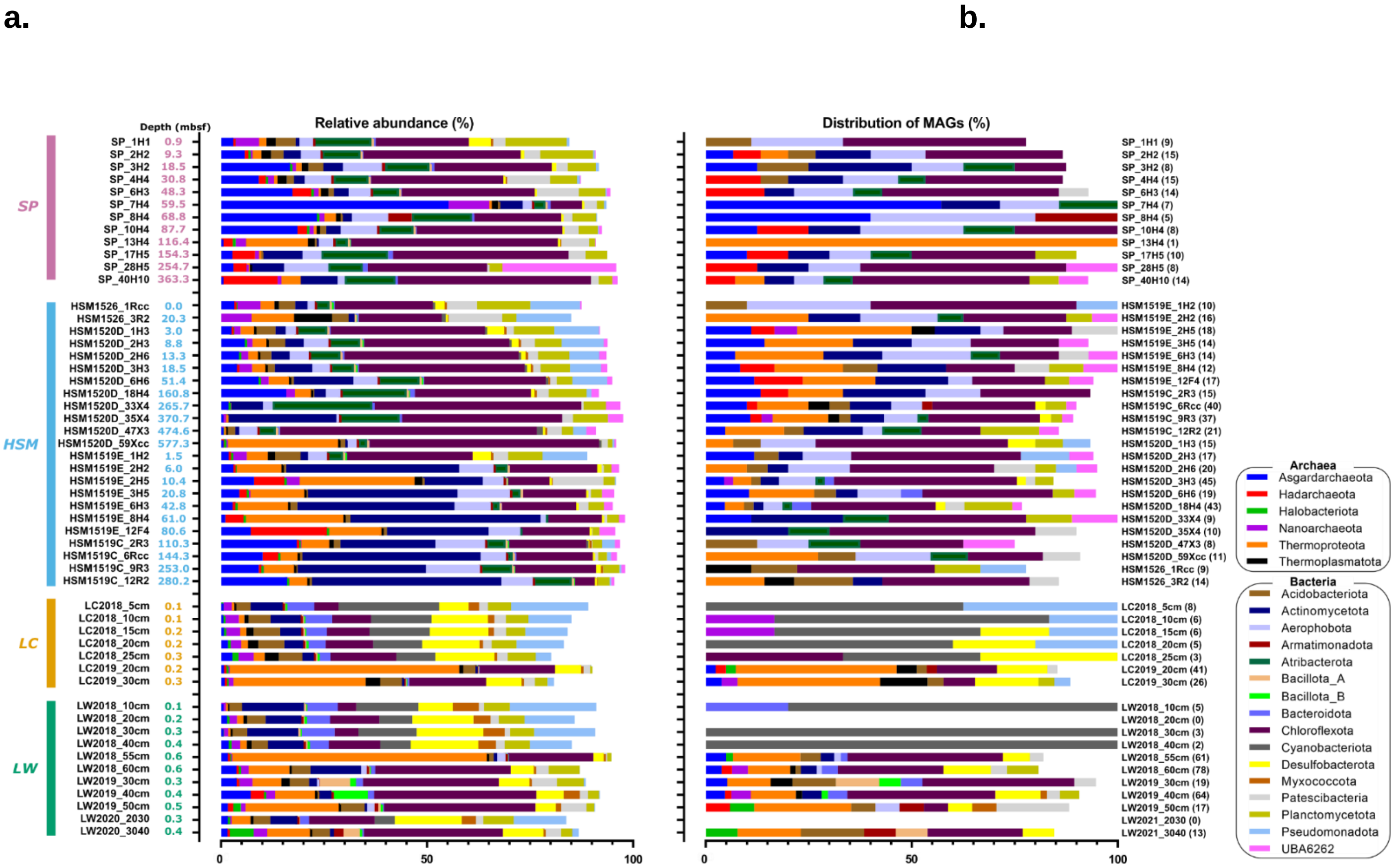

Figure S4 | Phylum-level community profiles and MAGs distribution of SP, HSM, LC and LW samples. **a.** Marker protein-based community profiles. Twenty-two abundant phyla with maximum relative abundances greater than 5% are shown in this figure. **b.** Taxonomic distribution of recovered medium-to-high quality MAGs. The total numbers of MAGs recovered in each sample were indicated in the brackets after the sample labels. For details, see Table S3 and S5 for community profiles, and Table S7 for MAGs. Figures were drawn with Prism9 (https://www.graphpad.com/)

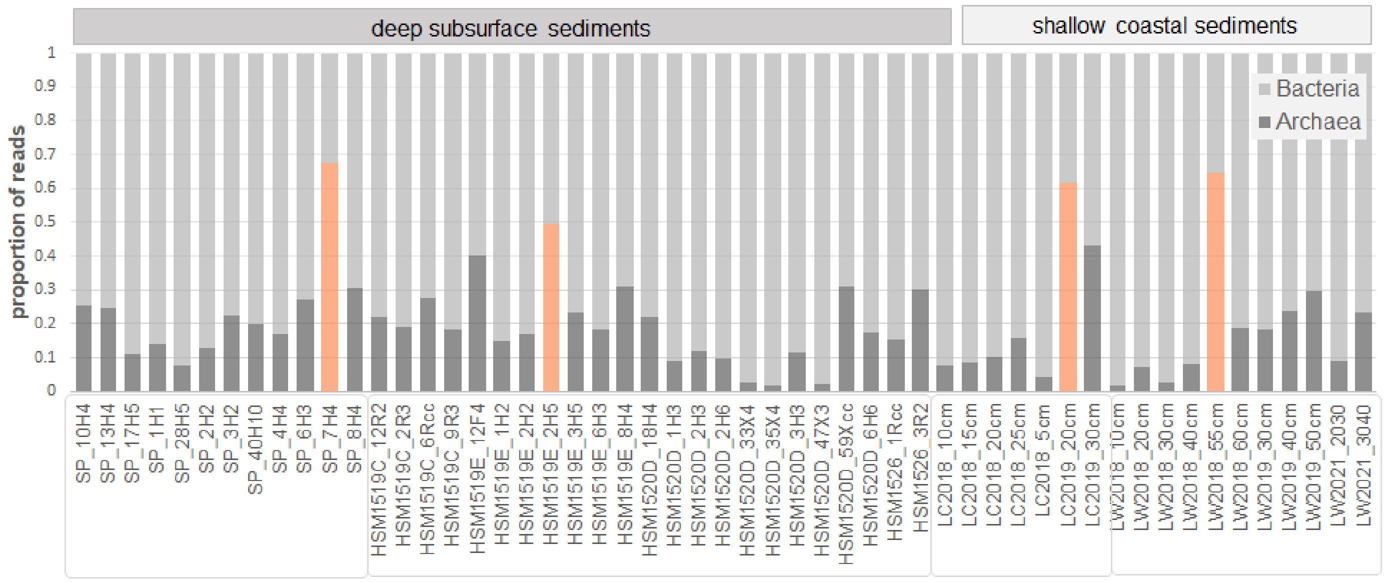

Figure S5 | Relative abundances of Bacterial and Archaeal domains. Relative abundances of Archaea and Bacteria across all samples are based on rarefied abundances from SingleM community profile.

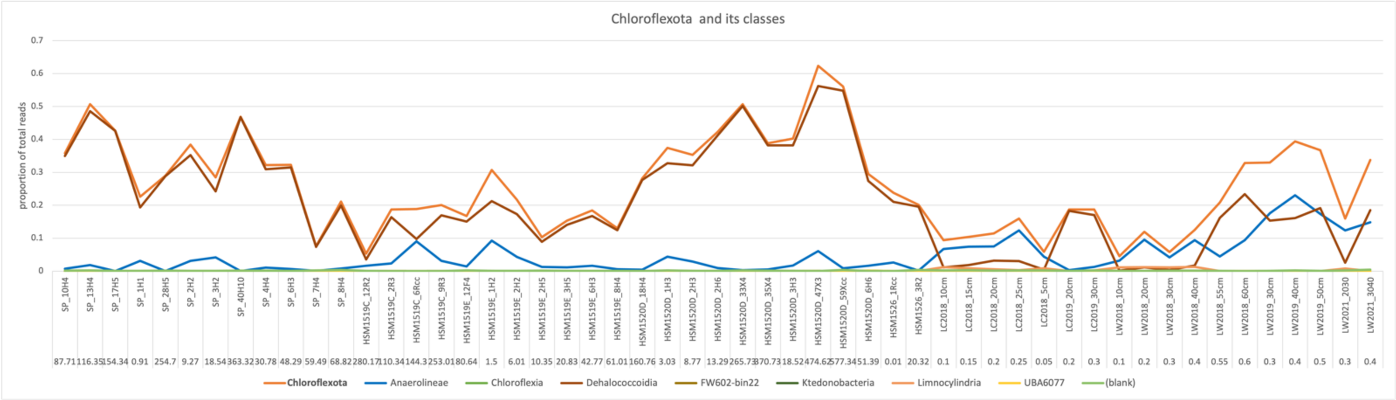

Figure S6 | Relative abundances of Chloroflexota and its classes. ‘blank’ represent unclassified Chloroflexota.

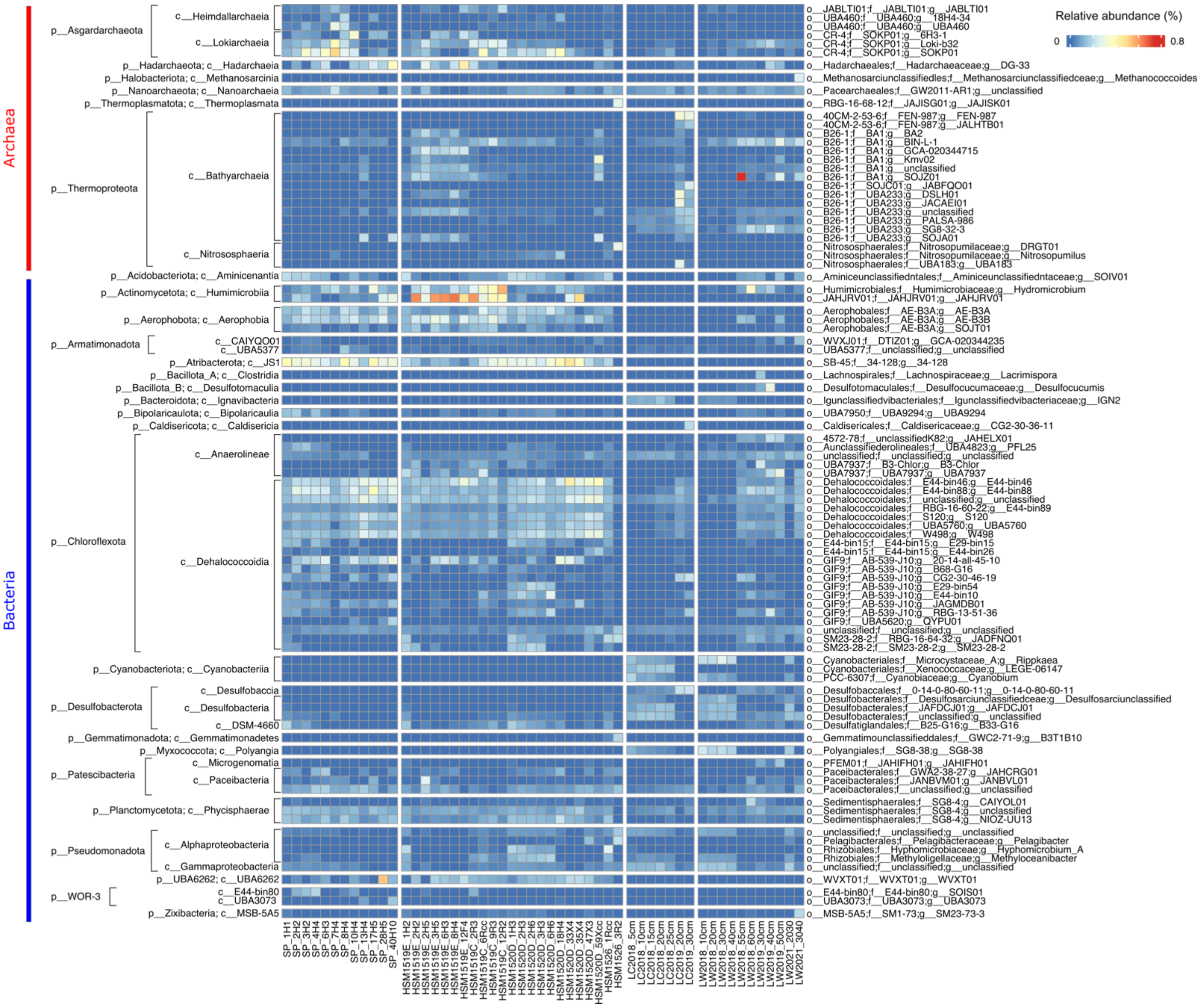

Figure S7 | Relative abundances of genera with maximum abundance > 2%. Relative abundances of mOTUs were first concatenated at genus level and transformed with square root.

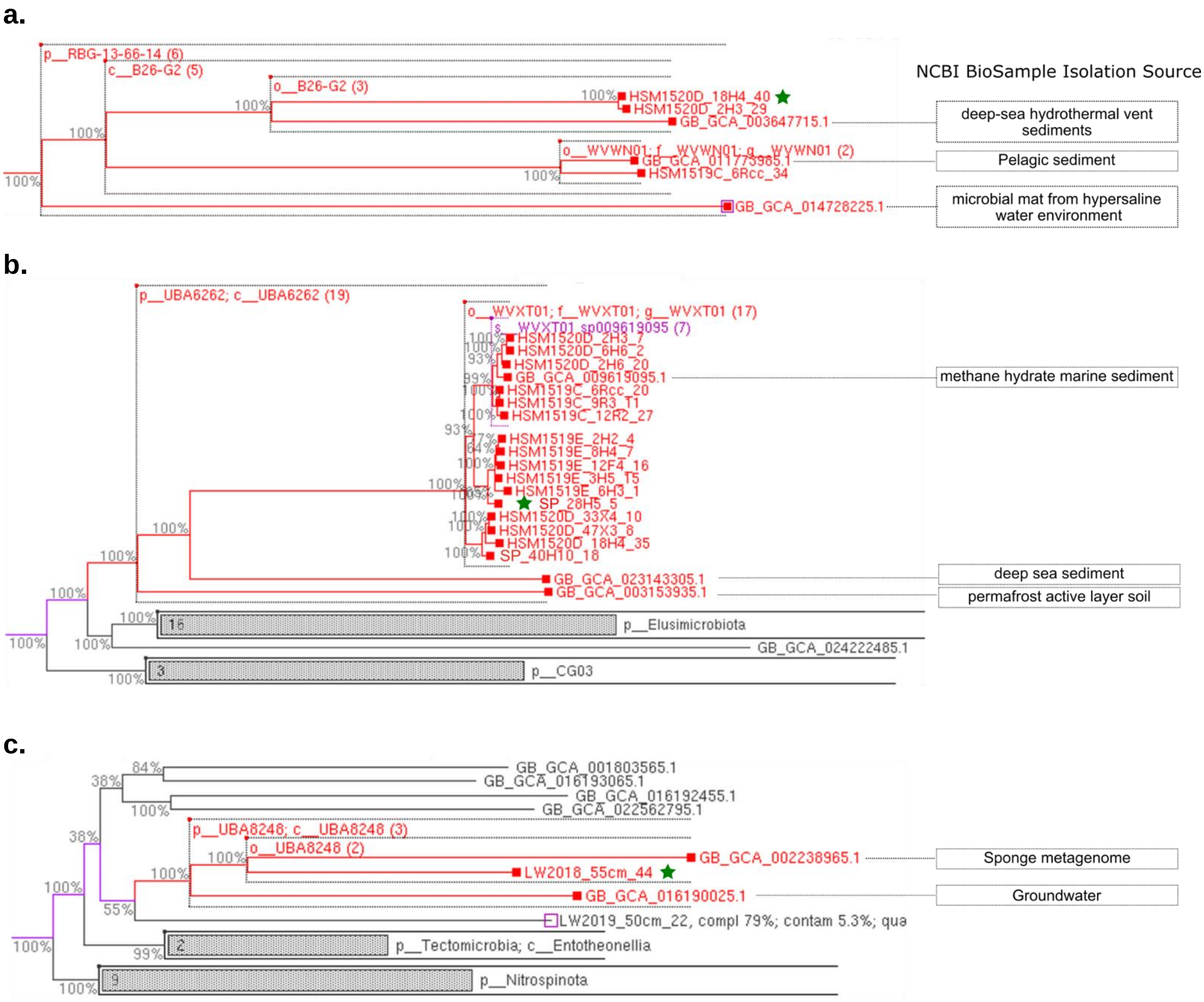

Figure S8 | Phylogenomics of novel phyla. a. RBG-13-66-14. Tangaroaeota, phyl. nov.; **b.** UBA6262. Ryujiniota phyl. nov.; **c.** UBA8248. Spongiisociota phyl. nov.. Trees were sections of a maximum-likelihood tree constructed with IQtree (LG+C10+F+G+PMSF, with 1000 ultrafast bootstraps) based on order level representatives of GTDB r214 taxa. Type genomes are marked with green star.

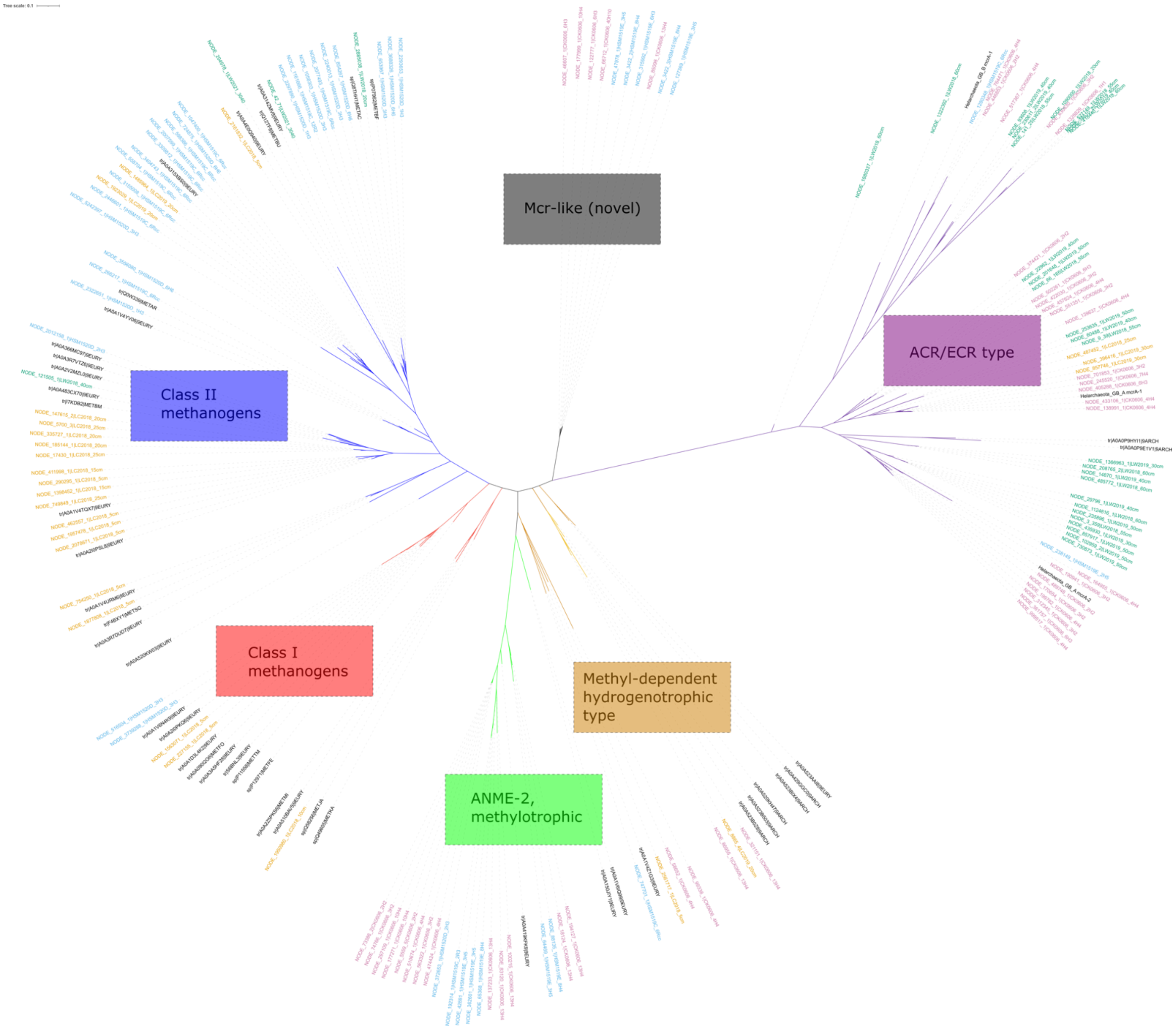

Figure S9 | Phylogenetic tree of McrA homologs from metagenomes. Maximum-likelihood analysis was performed using IQ-TREE with 1,000 ultrafast bootstraps under the LG+C10+F+G+PMSF model. Reference McrA sequences were labelled in black.

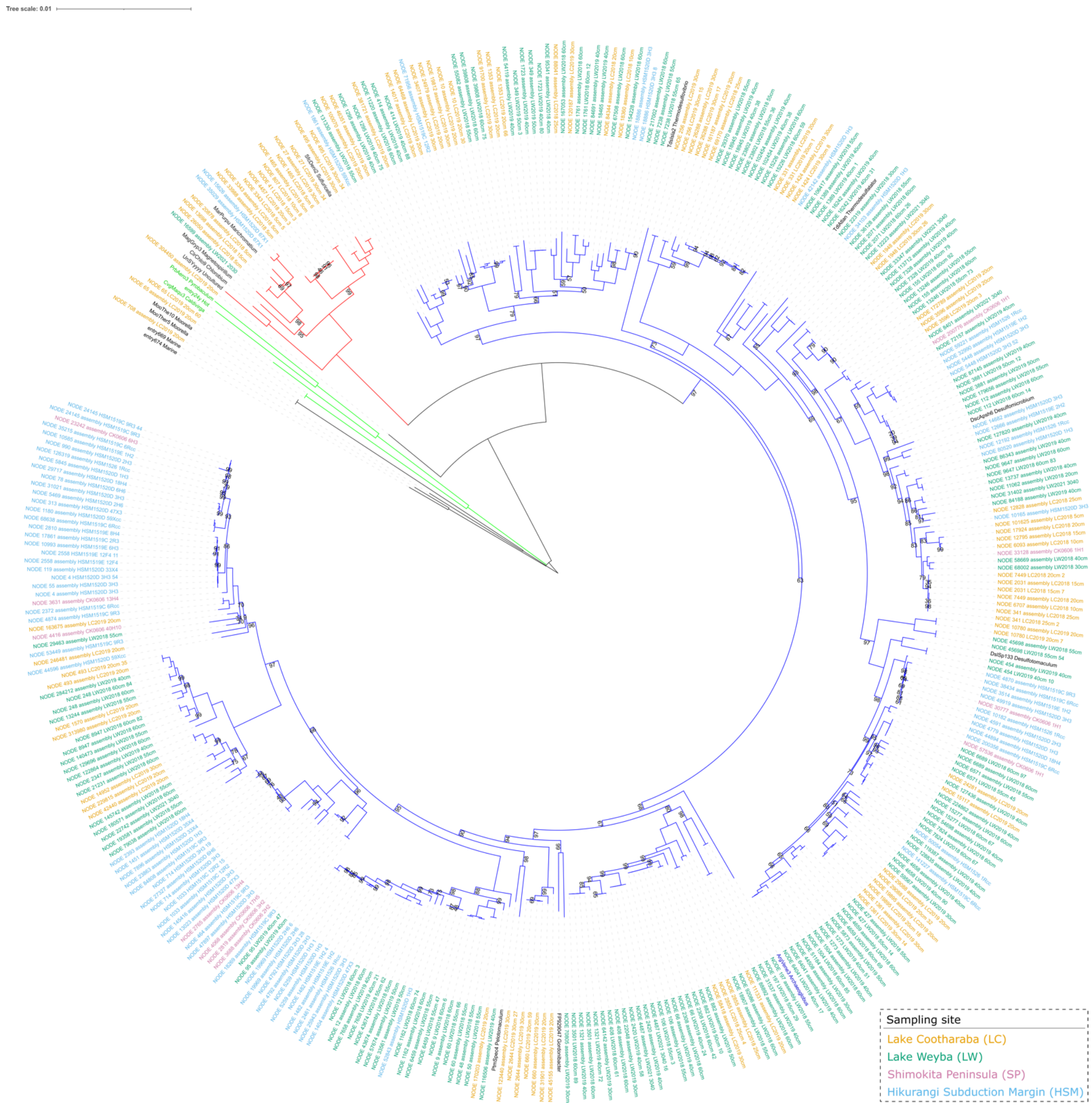

Figure S10 | Phylogenetic tree of DsrA homologs from metagenomes and MAGs. Maximum likelihood analysis was performed using IQ-TREE with 1,000 ultrafast bootstraps under the LG+C10+F+G+PMSF model. Three types of DsrA genes were found, including oxidative DsrA (with clade/branches coloured in red), reductive archaeal DsrA (with clade/branches coloured in green), and reductive bacterial DsrA (with clade/branches coloured in blue). The remaining sequences with black branches are unclassified DsrA. Colours of labels indicate the source sampling site of the dsrA gene sequence. Labels of reference DsrA are coloured in black.

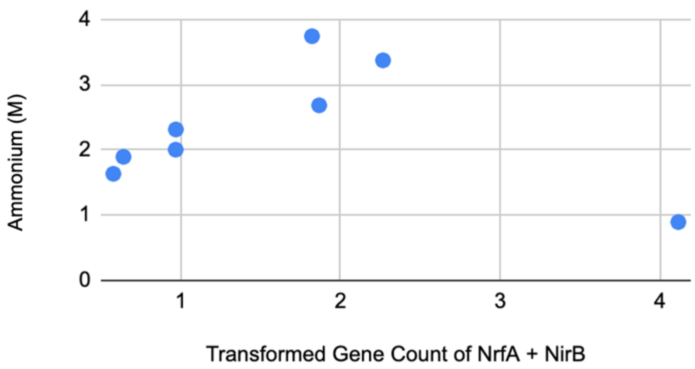

Sample ID	Depth (mbsf)	Source sample and depth	Ammonium (M)
HSM1520D_1H3	3.03	# 1H2, 2.93m	0.89
HSM1520D_2H3	8.77	# 2H2, 8.67m	1.63
HSM1520D_2H6	13.29	# 2H5, 13.19m	1.89
HSM1520D_3H3	18.52	# 3H2, 18.37m	2.31
HSM1520D_6H6	51.39	# 6H5, 51.24m	2.68
HSM1520D_18H4	160.76	# 18H3, 160.61m	3.74
HSM1520D_33X4	265.73	# 33X3, 265.53m	3.37

Figure S11 | Correlation of nitrite reduction gene counts and sediment ammonium concentration. Ammonium concentration of measured sediments and their depths are shown in the table at right. The correlation ($r = 0.896$, p-value = 0.006) was calculated by excluding one outlier: sample HSM1520D_1H3, obtained from 3.03 mbsf with the lowest ammonia concentration but the highest gene count.

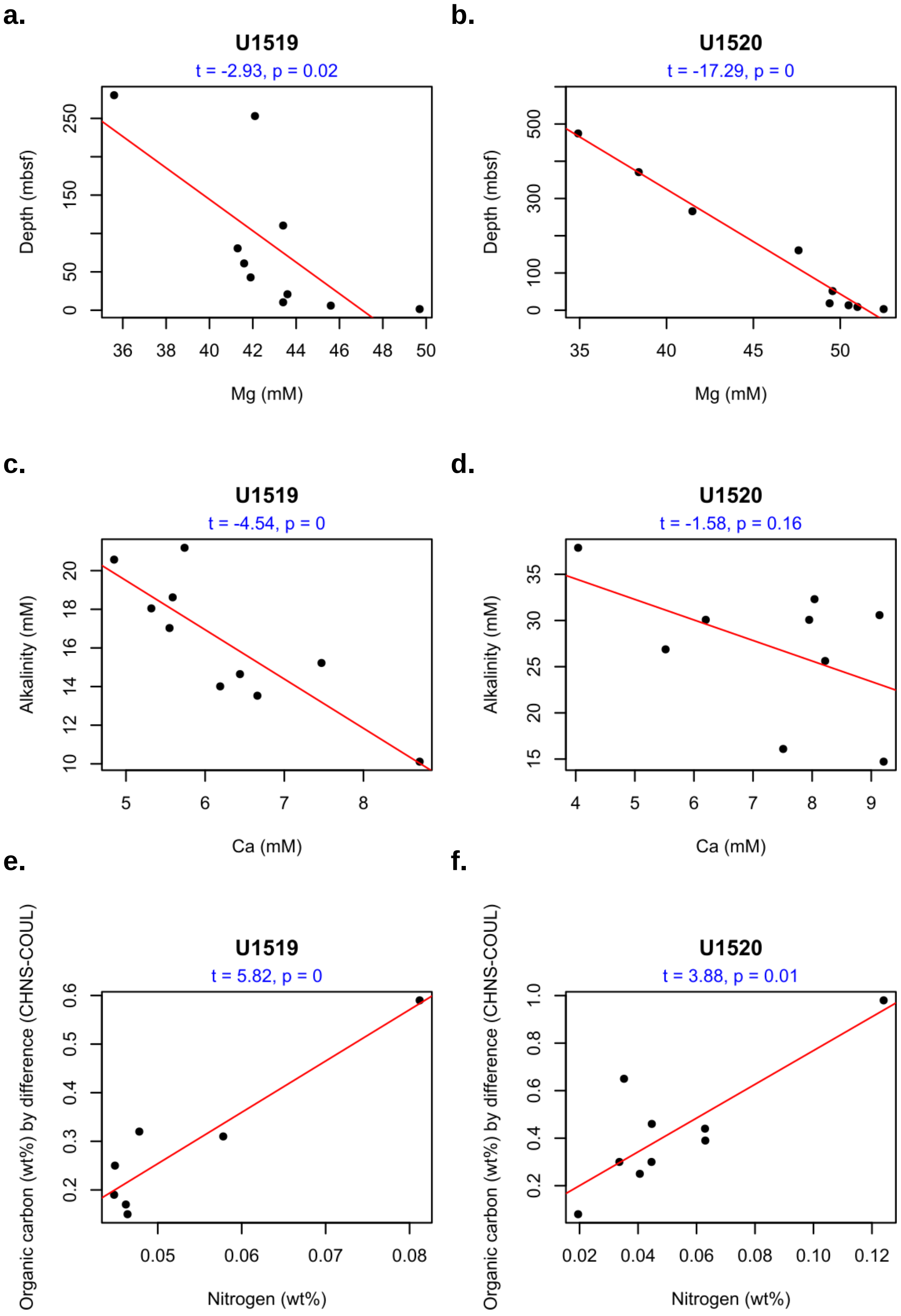

Figure S12 | Correlated chemical properties in analysed Hikurangi Subduction Margin sediments. a, b. Depth and concentrations of magnesium in sediments at HSM sites U1519 and U1520. **c, d.** Alkalinity and concentrations of calcium in sediments at HSM sites U1519 and U1520. **e, f.** Concentrations of organic carbon and nitrogen in sediments at HSM sites U1519 and U1520.
